# *un*Drift: A versatile software for fast offline SPM image drift correction

**DOI:** 10.3762/bjnano.14.101

**Published:** 2023-12-28

**Authors:** Tobias Dickbreder, Franziska Sabath, Lukas Höltkemeier, Ralf Bechstein, Angelika Kühnle

**Affiliations:** 1 Physical Chemistry I, Bielefeld University, Universitätsstraße 25, 33615 Bielefeld, Germany

**Keywords:** atomic force microscopy, calibration, drift correction, image correlation functions, periodic structures, scanning probe microscopy

## Abstract

Scanning probe microscopy (SPM) techniques are widely used to study the structure and properties of surfaces and interfaces across a variety of disciplines in chemistry and physics. One of the major artifacts in SPM is (thermal) drift, an unintended movement between sample and probe, which causes a distortion of the recorded SPM data. Literature holds a multitude of strategies to compensate for drift during the measurement (online drift correction) or afterwards (offline drift correction). With the currently available software tools, however, offline drift correction of SPM data is often a tedious and time-consuming task. This is particularly disadvantageous when analyzing long image series. Here, we present *un*Drift, an easy-to-use scientific software for fast and reliable drift correction of SPM images. *un*Drift provides three different algorithms to determine the drift velocity based on two consecutive SPM images. All algorithms can drift-correct the input data without any additional reference. The first semi-automatic drift correction algorithm analyzes the different distortion of periodic structures in two consecutive up and down (down and up) images, which enables *un*Drift to correct SPM images without stationary features or overlapping scan areas. The other two algorithms determine the drift velocity from the apparent movement of stationary features either by automatic evaluation of the cross-correlation image or based on positions identified manually by the user. We demonstrate the performance and reliability of *un*Drift using three challenging examples, namely images distorted by a very high drift velocity, only partly usable images, and images exhibiting an overall weak contrast. Moreover, we show that the semi-automatic analysis of periodic images can be applied to a long series containing hundreds of images measured at the calcite–water interface.

## Introduction

In science and technology, scanning probe microscopy (SPM) techniques are widely used to study the structure and properties of surfaces and interfaces from the micrometer scale down to the atomic level. The common element of SPM techniques is that surface structure and properties are revealed by moving a probe over the sample covering a given area or volume. During this movement, the interaction between probe and sample is measured at fixed points in the scan area resulting in an image of the structure or a specific surface property. This scanning process is also the origin of two prominent artifacts in SPM image data, that is, an imperfect scanner calibration and thermal drift. Both of which cause a misalignment between probe and surface; thus, the measured SPM images are distorted and potentially shifted [1–6].

The calibration of an SPM scanner is used by the SPM instrument to convert the voltages applied to the scanner piezo into a probe position [2,7]. If this calibration is not correct, the position assumed by the SPM instrument deviates from the actual probe position; thus, the SPM image is distorted [1–2,4,8–17]. The determination of linear calibration parameters and scanner non-orthogonality based on atomic structures [1–3,8–9,18–19] and calibration gratings [1,20–22] have been discussed intensively. In addition to linear effects, non-linear scanner behavior such as creep [1,4,10–13] and hysteresis [1–2,4,10,14–17] has been analyzed and corrected.

The second prominent artifact causing distortion of SPM images is (thermal) drift. Because of the serial nature of the scanning process, recording an entire image takes at least several milliseconds in case of video-rate scanning [23–24]. Most SPM images, however, are measured at a much lower scan rate on the timescale of seconds to minutes. During this measurement time, the temperature in the SPM instrument can fluctuate, inducing thermal expansion or contraction of the instrument’s components [5]. As a consequence, sample and probe experience an unintended movement relative to each other, that is, the thermal drift. This drift is not included in the measurement data, so the recorded SPM images appear distorted [5–6]. An effective way to reduce thermal drift to a minimum is to carry out SPM experiments under cryogenic conditions close to the temperature of liquid helium. The cryogenic temperature, however, also drastically reduces the rates of thermal processes such as on-surface reactions, diffusion, or desorption [25]. Hence, many processes relevant at room temperature or elevated temperatures are impossible to study in a cryogenic environment. The same applies to SPM studies at the solid–liquid interface. For these measurements, the effect of thermal drift needs to be compensated.

A variety of different strategies have been developed to characterize and correct thermal drift in SPM measurements. There are two types of drift correction strategies: In online drift correction, the drift velocity is determined during the measurement; then, the scanner movement is adjusted to compensate for drift [16,25–28]. Offline drift correction strategies, in contrast, correct the effect of drift in SPM images after the measurement. Drift correction has been carried out based on the apparent movement of stationary features (e.g., fixed defects or adsorbates) traceable in consecutive images [5,29–31] or images with opposing scan directions [12,32]. Instead of analyzing the apparent shift of individual features, the apparent movement of the scan window can also be determined from the maximum of the cross-correlation [26–27,33–36] between consecutive images. Moreover, the different distortion of periodic structures in images with opposing scan directions was used to determine drift and to calculate the undistorted structure [3,37]. Other authors proposed drift correction procedures based on rescanning a small area of an SPM image with the fast and slow scan directions reversed [13,38–40], splitting the scan of an image into several subscans [41], or periodically rescanning the first scan line [42]. For non-raster SPM, the drift velocity can be extracted without additional scans from the analysis of inherent crossing points in the scanning path [43–44]. Another approach to remove distortions from SPM images is to correct the images with regard to a known reference structure [4,6,18]. While the latter strategy can ensure distortion-free SPM images for known surface structures, it is not suited for investigation of unknown structures.

Here, we present *un*Drift, a free-to-use scientific software for the fast and reliable calibration and drift correction of SPM image data. *un*Drift implements three algorithms to determine the drift velocity based on two consecutive SPM images with or without periodic structures. The first semi-automatic algorithm extracts lattice vectors from two consecutive up and down (down and up) images exhibiting periodic structures. These lattice vectors are, then, used to analyze the distortion of the images and to calculate the drift velocity. This algorithm enables *un*Drift to drift-correct both SPM images without stationary features and SPM images without overlapping scan areas. The second and third drift correction algorithms extract drift velocities from the apparent movement of stationary features as described in [5,12,25–27,29–36,45]. Algorithm II implements the well-known cross-correlation method [26–27,33–36,45] to automatically determine the shift between two SPM images with identical scan directions. From this shift, we calculate the drift velocity. Algorithm III, in contrast, provides the possibility to manually identify stationary features in two SPM images with arbitrary scan direction and use their positions for drift correction [5,12,25,29–32]. Note that all drift correction algorithms applied by *un*Drift rely on information contained within the measurement data solely; thus, *un*Drift allows for the investigation of unknown structures. To demonstrate the performance of *un*Drift, we apply our software to three examples where the drift correction is especially challenging. Namely, we drift-correct SPM images with a very high drift velocity exceeding the slow scan rate, only partly usable images, and images with an overall weak contrast and high noise level. Moreover, we show that *un*Drift is suitable for the drift correction and evaluation of measurement sessions spanning several hundred images.

## Software

*un*Drift is scientific software for the fast and accurate drift correction and calibration of SPM image data as necessary for quantitative data analysis. It is written in JavaScript and HTML and runs with all common browsers independent of the operating system. *un*Drift can be operated either on a local server or as a web-based version. Both versions are available from our website under [46]. All functions of *un*Drift, such as data import, leveling, calibration, and drift correction of SPM data, are free to use, that is, *un*Drift is entirely free to use. Analysis results including corrected SPM images as well as extracted lattice vectors and drift velocities are available in standard open data formats.

The full source code of *un*Drift is also available from our website. JavaScript modules for the import and analysis of SPM data are licensed under the GNU General Public License version 3.0. The visualization of SPM images in *un*Drift is realized with the proprietary library Kontrast [47], which is available under its own license. This means that all functions of *un*Drift are free-to-use for non-commercial purposes but the development of new features requires a Kontrast license.

### Input data

The import of SPM data into *un*Drift is designed to read files in the Gwyddion Native Format (gwy format) as created with the open source SPM data analysis software Gwyddion [48]. Gwyddion contains import modules for a wide variety of scanning probe microscopes as well as tools for SPM data processing and analysis. This enables the user to chose between data processing in *un*Drift and Gwyddion. *un*Drift supports basic data processing methods such as leveling (mean plane, polynomial) and an automatic color scale adaption; thus, the processing of most standard images can be done directly in *un*Drift. For more complex processing steps, the user is referred to Gwyddion. Then, the pre-processed SPM data can be calibrated and drift-corrected with *un*Drift. Moreover, *un*Drift can export data in the gwy format, which enables a seamless integration between *un*Drift and Gwyddion.

The gwy format does not contain standardized containers for scan direction, scan angle, and raster time per pixel, which constitute vital information for an accurate drift correction and calibration. *un*Drift extracts these parameters from the metadata, where they are typically stored with varying names depending on the manufacturer and version of the used microscope. Because of this, it is necessary to specify the matching between scanning parameters and metadata names for each microscope individually. We did this specification for the instruments and file formats listed in Table 1. For all other devices, this easy step needs to be done by the user in the preferences of *un*Drift.

**Table 1:** Overview of SPM devices and third-party file formats supported by *un*Drift.

Device	Format	Direct import	Import via Gwyddion

Omicron MATRIX	.Amplitude_mtrx, .Damping_mtrx, .Phase_mtrx, .Df_mtrx, .Z_mtrx	no	yes
Bruker Nanoscope V	.001, .002, …	no	yes
Cypher ES AFM	.ibw	yes	yes
Nanonis controller	.sxm	yes	yes
other devices supported by Gwyddion		no	limited^a^
others		no	no

^a^In principle, *un*Drift can read all SPM data saved with Gwyddion in the gwy format. However, as the gwy format lacks standardized containers for some of the information necessary for drift correction and calibration, the user needs to specify the mapping of meta data headers in the preferences of *un*Drift before use.

### Drift correction

Next, we will discuss the main feature of *un*Drift, the offline drift correction of SPM images. Depending on the surface structure and scan directions of the SPM images, *un*Drift provides different algorithms to determine the drift velocity and to drift-correct the data. In terms of surface structure, we distinguish between images exhibiting a periodic structure and those without periodic structures. For images showing two-dimensional periodic structures, the drift velocity can be calculated from the different distortion of the surface periodicity in two SPM images with opposing slow scan direction (algorithm I). For the drift correction of SPM images without periodic structures, in contrast, *un*Drift implements two strategies (algorithms II and III) described in literature [5,12,25–27,29–36,45] to extract the drift velocity from the apparent movement of stationary features in consecutive images. Algorithm II uses the cross-correlation function between two images recorded in the same scan direction to evaluate the drift velocity [26–27,33–36,45], while algorithm III evaluates the position shift of stationary features between two images with arbitrary scan direction [5,12,25,29–32]. Both algorithms II and III also work for images exhibiting periodic structures if they contain stationary features. In the following two sections, we discuss the different algorithms clustered by the surface structures they are suitable for. Regardless of surface structure and scan direction, however, all drift correction algorithms applied by *un*Drift rely on information contained within the measurement data solely; thus, they allow for a quantitative data analysis without any knowledge on the surface geometry. Instead, all of the applied algorithms use the difference between two consecutive images to determine the drift velocity and to correct the SPM data. Hence, *un*Drift is, in contrast to drift correction schemes relying on known surface geometries, suited to investigate substrates with an unknown surface structure.

#### Images with periodic structures

**Figure 1:**
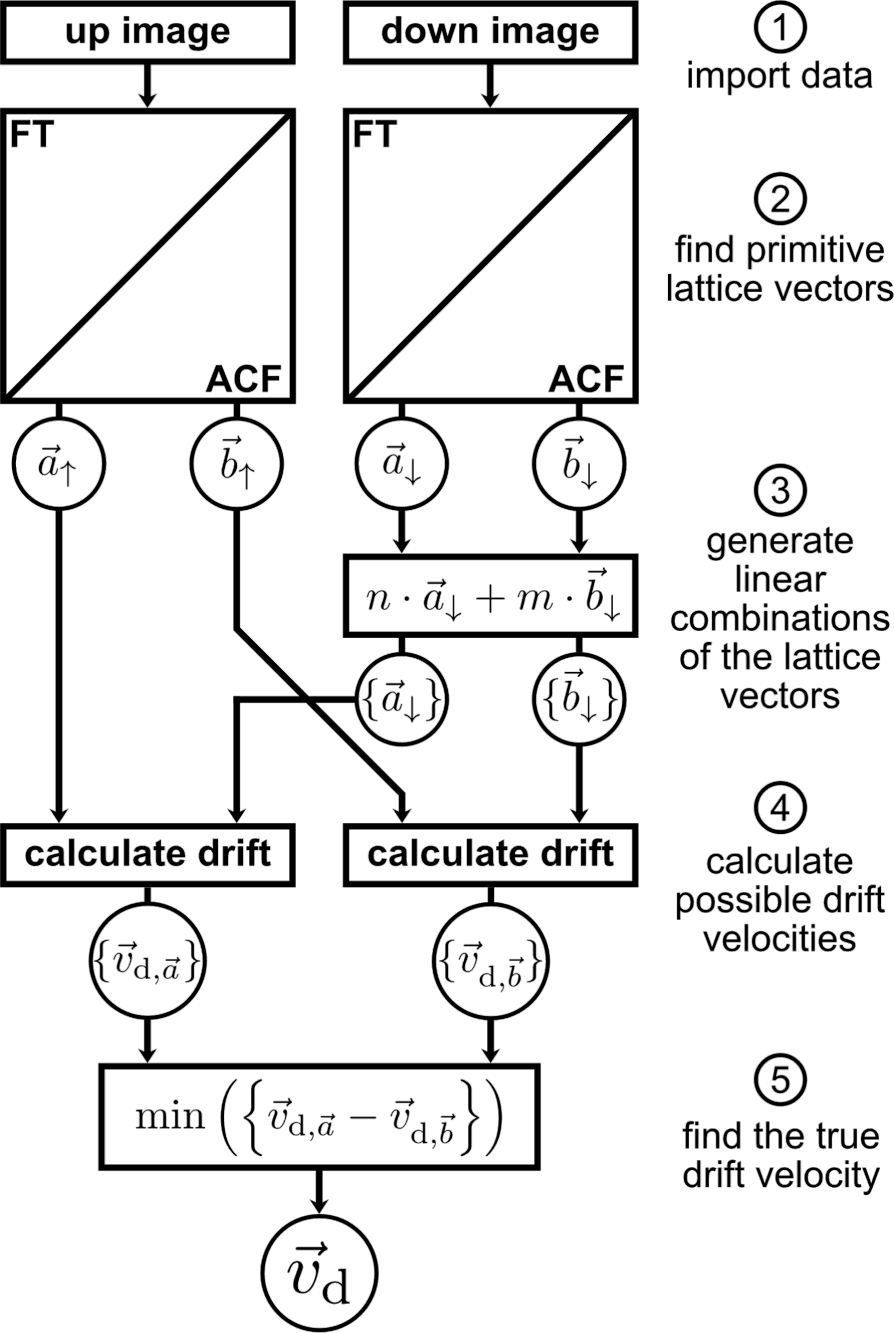
Schematic representation of algorithm I used to determine the drift velocity *v*_d_ from the distortion of periodic structures in consecutive SPM images with opposing slow scan directions.

**Figure 2:**
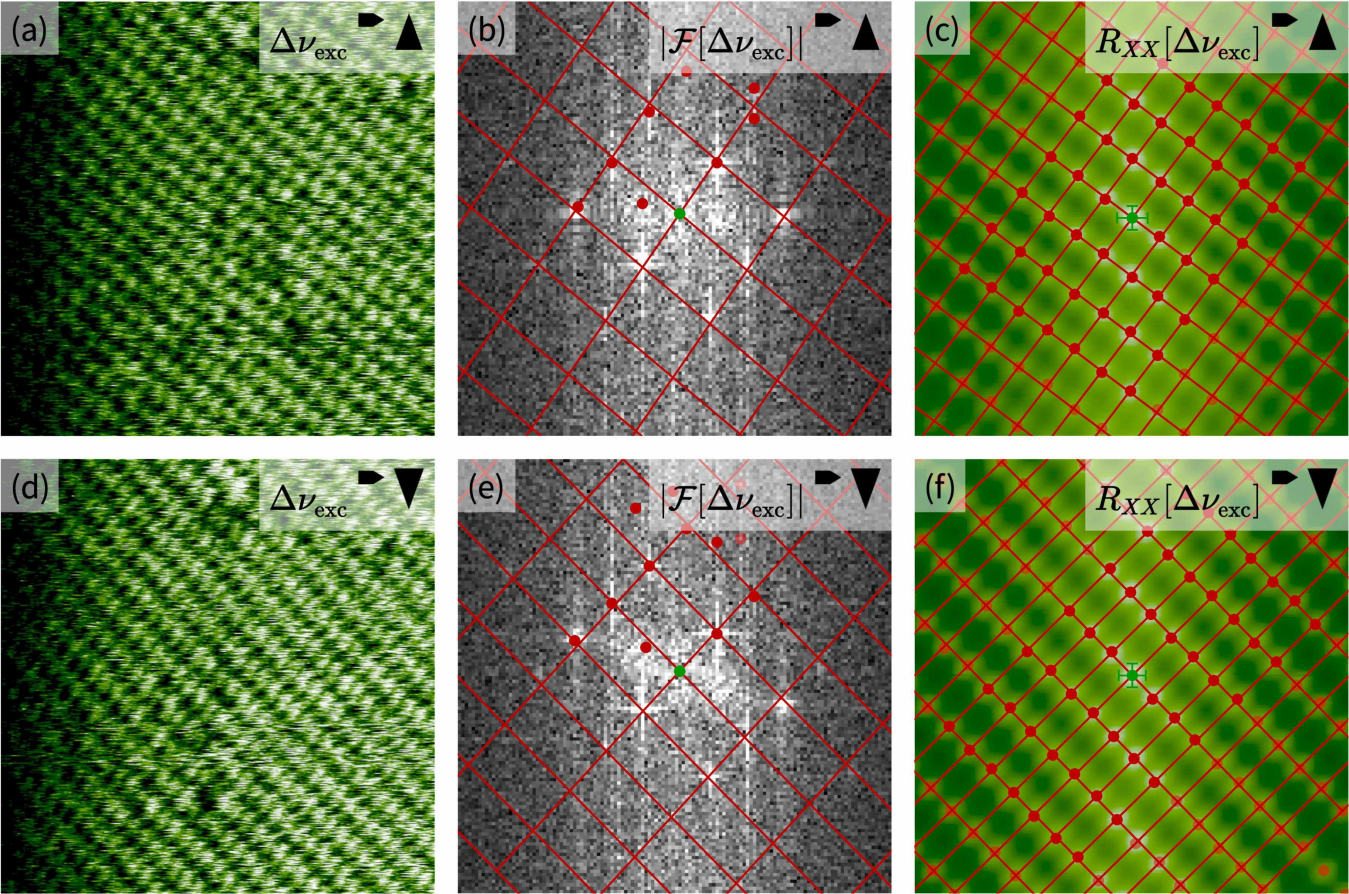
Extraction of lattice vectors from images exhibiting periodic structures. (a, d) High-resolution AFM images showing atomic resolution at the calcite (10.4)–water interface. (b, e) Fourier transform images of the real-space images shown in (a) and (d). The maxima in the Fourier transforms are marked by red circles, and the optimized lattice as found by *un*Drift is shown as red lines. Only the centers of the Fourier transformations are shown. (c, f) Autocorrelation images of the real-space images shown in (a) and (d). The maxima in the autocorrelation images are marked by red circles, and the optimized lattices as found by *un*Drift are shown as red lines. Only the centers of the autocorrelations are shown.

For the drift correction of images exhibiting periodic structures, *un*Drift uses the algorithm shown in Figure 1 (algorithm I). This algorithm takes advantage of the characteristic distortion of periodic structures in SPM images depending on drift velocity and scan direction [3,6,12,37]. For two consecutive SPM images with opposing slow scan directions, we will observe two different apparent surface structures, as drift distorts the real periodic surface structure differently for a different scan direction (see Figure 2a,d). As we know that the real surface structure is, indeed, independent of the scan direction, we can use the difference between distortions to calculate the drift velocity (and the real surface structure) as described in Supporting Information File 1. Algorithm I has the advantage that it relies only on the surface periodicity; thus, the scan areas of the two images used for drift correction do not need to overlap. The only requirements for this algorithm are a constant drift velocity and two SPM images exhibiting periodic structures measured with different slow scan directions. Note that, in principle, this strategy is not limited to images with opposing slow scan directions but should also work for images with different fast scan directions. In real measurements, however, we find that the difference in the distortion of images with different fast scan directions is too small compared to the uncertainty to achieve reliable results for the drift correction. Consequently, *un*Drift implements this algorithm for consecutive images with opposing slow scan directions solely.

As shown in Figure 1, algorithm I consists of five main steps, namely (1) import of input data, (2) extraction of primitive lattice vectors, (3) generation of linear combinations of these lattice vectors, (4) calculation of possible drift velocities, and (5) selection of the true drift velocity. After that, the obtained drift velocity is used to calculate the real lattice vectors and drift-correct the SPM images.

First, two SPM images with opposing slow scan directions (i.e., one up and one down image) are imported as described before (step 1). Next, *un*Drift extracts primitive lattice vectors from both images as shown in Figure 2 (step 2). *un*Drift can determine the lattice vectors either based on the Fourier transforms (Figure 2b,e) or autocorrelations (Figure 2c,f). In both cases, our software searches for local maxima in the transformed image first (red dots in Figure 2). Then, it extracts guesses for the lattice vectors based on the maxima with the highest intensity, the maxima closest to the origin, or based on user selection. After that, these guessed lattice parameters and the spots belonging to the lattice are optimized with a least squares algorithm to obtain optimal lattice parameters. In Figure 2 the optimized lattice parameters are shown as a lattice drawn with red lines. For the Fourier transform, this fit yields lattice vectors of the inverse lattice, which are then transformed into real-space lattice vectors. For the autocorrelation, the optimization yields the real-space lattice vectors directly. To finish step 2, *un*Drift applies simple geometry to calculate the shortest possible lattice vectors for the lattice extracted in the optimization step.

This extraction of lattice vectors is a semi-automatic step, as the user needs to specify parameters for the peak finding. While these parameters need to be chosen manually, it can be easily determined from the fit quality whether these parameters are chosen correctly. We find that the optimal parameters for this step depend on the image size and contrast; thus, it can be necessary to change these parameters after a contrast change. Moreover, the user needs to choose whether the algorithm operates based on Fourier transform or autocorrelation. This choice mostly depends on the size of the image relative to the size of the observed periodic structure. For images showing many unit cells, we find that the Fourier transform yields better results, while the autocorrelation is superior for images containing very few unit cells. In between these extremes, there is a range where both methods work well as shown in our examples in Figure 2. The dependence of the optimal transformation for peak extraction on the image size is caused by the finite resolution of the measured SPM images. For images containing many unit cells, each unit cell only consists of few pixels; thus, the maxima in the autocorrelation are difficult to separate, as they also consist of few pixels. The Fourier transform, in contrast, works in the inverse space, which is why the maxima are very nicely separated by many pixels and easy to find by the algorithm. For images containing few unit cells, it is the other way around. The autocorrelation maxima are nicely separated, while the maxima in the Fourier transform sometimes even merge together.

After the extraction of lattice vectors, *un*Drift applies steps 3 to 5 to determine the drift velocity from these lattice vectors. It is important to note that the calculation of drift velocities requires a set of two differently distorted versions of the same vector (see Supporting Information File 1). The identification of a matching pair of lattice vectors, however, becomes increasingly difficult with increasing drift velocity and, thus, increasing image distortion. To solve this problem and to ensure that *un*Drift also works reliably for high drift velocities, we apply steps 3 to 5 of algorithm I. The idea behind this part of the algorithm is the following: Each pair of lattice vectors (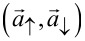 and 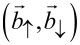) yields one drift velocity. Hence, we can calculate two seemingly independent drift velocities 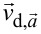 and 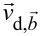. In reality, however, there is only one drift velocity 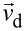 that applies for both images. Hence, the drift velocities calculated for both lattice vectors need to be identical, that is, 
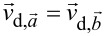
.

Next, we can use this criterion to find matching pairs of lattice vectors and, thus, calculate the true drift velocity. We can start by choosing a set of lattice vectors for the first image, as this initial choice is arbitrary. For the second image, however, we need to find the matching lattice vectors. To realize this, we take the lattice vectors extracted from the image and generate a set of linear combinations for both vectors 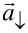 and 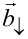 (see Figure 1, step 3). These sets 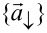 and 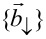 then contain the matching vectors as well as several other vectors. In the next steps, the program subsequently calculates the corresponding sets of drift velocities 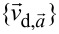 and 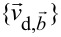 (step 4) and selects the pair of drift velocities with the smallest difference between 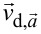 and 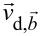 (step 5). Note that we cannot search for identical drift velocities, as the individual drift velocities are always slightly different because of experimental noise and a small non-linearity in the drift. Instead, we use the two drift velocities with the smallest difference. Finally, the true drift velocity 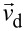 is calculated by averaging over the individual drift velocities.

For the selection of the drift velocity in step 5, *un*Drift provides an alternative selection procedure based on the lattice vectors. Not only the drift velocity needs to be identical for both pairs of lattice vectors, but also the primitive unit cell with the shortest lattice vectors has to be identical in both images after drift correction. The second selection procedure, thus, operates based on the difference between drift-corrected lattice vectors in both images. Again, we search for the minimum difference and select the corresponding drift velocity as the real drift velocity. The selection procedure used in step 5 can be chosen by the user under lattice matching. We find that this second selection procedure tends to work better for images with low signal-to-noise ratio.

#### Images without periodic structures

As discussed before, *un*Drift features two algorithms to drift-correct SPM images without periodic structures based on the apparent movement of stationary features in consecutive images. In this section, we will describe these two algorithms, referred to as algorithm II and III, and discuss their applicability to different situations in terms of scan directions and experimental systems.

**Figure 3:**
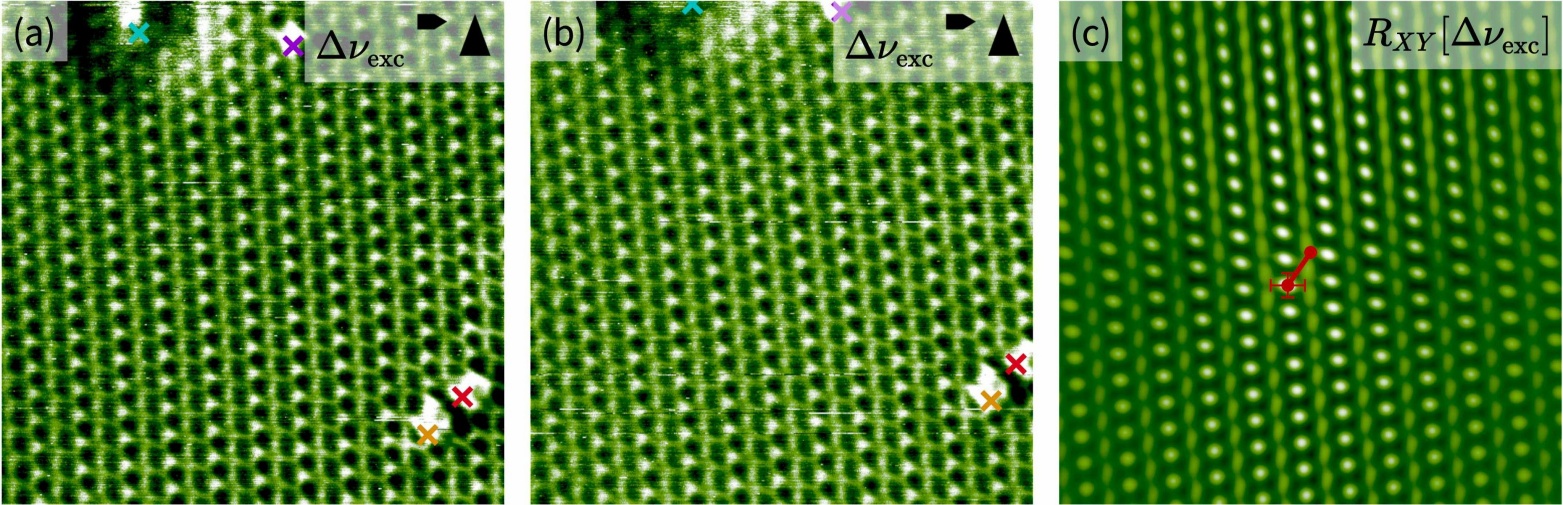
(a, b) Two consecutive up images recorded with high-resolution AFM on calcite(10.4) in ultrahigh vacuum. The images show several defects, whose positions are marked with colored crosses in both images. It is apparent that the defect positions are shifted in the second image compared to the first one. (c) Image of the cross-correlation function *R_XY_* between the images shown in (a) and (b). The center of the image and the position of the maximum are marked with red dots and connected with a red line. This line corresponds to the shift between images (a) and (b).

Algorithm II uses the cross-correlation between two consecutive images recorded in the same scan direction to evaluate the shift between the images and to calculate the drift velocity. The distortion of SPM images by drift only depends on drift velocity and scan direction (see Supporting Information File 1). Hence, two images measured with the same scan direction and a constant drift velocity are distorted in the same way. The only difference between these two images is that they are measured at a slightly different position on the surface, because drift moved the scanner and surface relative to each other. In SPM images, this effect manifests itself in an apparent movement of stationary surface features whose positions are actually constant, such as defects and step edges. This is illustrated in Figure 3, where the stationary surface features marked with colored crosses seem to move between Figure 3a and Figure 3b. Here, it is important to note that all stationary features “moved” by the same vector.

To evaluate this shift between images, we can apply the cross-correlation function between both images as described in [26–27,33–36,45]. In the cross-correlation image, the global maximum corresponds to the shift necessary to achieve maximum similarity between both images [33–34], where a maximum in the image center means that both images are identical without any shift. We show the cross-correlation of our two example images (Figure 3a,b) in Figure 3c. We marked the center of the image and the global maximum with red dots connected by a red line to illustrate the shift between images. When we compare the shift derived from the cross-correlation image, it is evident that this shift is identical to the shift of the stationary features between the images in Figure 3a and Figure 3b.

In the next step, *un*Drift uses the shift derived from the cross-correlation function to calculate the drift velocity as described in Supporting Information File 1 and to drift-correct the input data. This algorithm is fully automatic and does not require any additional input from the user.

In addition to the global maximum, the cross-correlation image in our example in Figure 3c also exhibits a periodic structure. This periodicity stems from the periodic structure observed in the input images, and it can be evaluated by *un*Drift to obtain lattice parameters. For the drift correction with algorithm II, however, the stationary features in the input images are decisive and not the surface periodicity. This algorithm does not work reliably for perfectly periodic surfaces without any stationary features as all maxima would have the same intensity; thus, the algorithm cannot decide which maximum corresponds to the shift between the images [30]. For images exhibiting a periodic structure and stationary features, however, we find that algorithm II works with remarkable reliability.

In contrast to algorithm II, algorithm III does not evaluate the mean shift between images but the apparent shift of single stationary features as described by Rahe and co-workers [5]. It is necessary to subsequently identify stationary features in the SPM images and to determine their positions. For the determination of a feature position, *un*Drift provides the possibility to refine the manually selected positions to the closest maximum or minimum. The drift velocity is calculated for each feature individually; then, the mean drift velocity can be calculated by averaging over the individual drift velocities. The deviation between these yields information about the non-linearity of the drift. With algorithm III, *un*Drift provides the possibility to use positions of stationary features identified manually by the user. The software displays both images used for drift correction and a variable number of markers in different colors as shown in Figure 3a,b. The user can, then, move these markers to match the positions of stationary features and start the drift correction by clicking a button. As this positioning of markers on identical positions of the stationary features is never perfect, we recommend to use as many stationary features as possible for the drift correction.

Algorithm III is arguably the slowest algorithm in *un*Drift in terms of evaluation time per image, and the selection of feature positions is, at least, partly subjective. However, algorithm III has the important advantage that it works for all images with stationary features regardless of scan direction and image quality. As long as the user can identify features in the SPM images, this algorithm will work for the drift correction. Moreover, algorithm III offers the possibility to easily assess and quantify the effect of non-linear drift in the drift-corrected SPM data, which is not available from the other algorithms.

### Lateral calibration

In addition to the correction of drift, it is crucial for a quantitative analysis of SPM data that the scanner position is calibrated properly. While the drift correction will remove the effect of additional movement between scanner and sample surface, the obtained positions, distances, and angles will still be incorrect as long as the instrument is not properly calibrated. This situation is well known in literature, and there are several different strategies documented to determine the calibration parameters for a scanning probe microscope [1–3,7–9,12,18–19,21–22]. With these calibration parameters, the microscope can either be calibrated before the measurement, or the measured SPM data can be corrected afterwards. The calibration parameters can change over time because of, for example, piezo aging or measurements at different temperatures [3,20]. Thus, it is necessary to validate the calibration parameters regularly. *un*Drift provides features to (1) correct SPM data with a given set of lateral calibration parameters and (2) determine lateral calibration parameters based on a reference surface geometry.

The lateral calibration of SPM data in *un*Drift is based on the assumption of a linear relationship [3,7–9,19] between recorded and actual scanner movement as described in Supporting Information File 1. In this case, the calibration only depends on three parameters, namely the correction factors in the *x* and *y* directions, κ*_x_* and κ*_y_*, respectively, and the angle between both directions, β. These parameters can be provided by the user for a lateral calibration of the drift-corrected SPM data. Alternatively, the default values can be used, which correspond to no additional calibration.

For SPM images exhibiting a periodic structure, *un*Drift can also calculate the expected lateral calibration parameters based on a reference. In order to do so, the user needs to provide the lattice parameters of the investigated periodic structure, that is, the edge lengths and opening angle of the surface unit cell. *un*Drift then compares these reference data to the measured lattice parameters and calculates the lateral calibration parameters necessary to match the measured periodicity with the reference. Thus, *un*Drift makes it very easy to calibrate a previously not calibrated device or to check whether an existing calibration is still valid. Detailed information on the determination of calibration parameters are given in Supporting Information File 1.

### Output data

After calibration and drift correction, *un*Drift provides a variety of different methods to export the obtained data as shown in Table 2. Corrected SPM images including Fourier transforms and autocorrelations are available for export in the Gwyddion Simple Field and Gwyddion Native formats for further data evaluation or processing in Gwyddion. Moreover, images and image cutouts can be exported into the standard image formats png and svg. The svg exporter also includes the possibility to export ready-to-use figures with annotations and scale bars.

**Table 2:** Overview of output data and export types available from *un*Drift.

Data type	Image type	Output formats

corrected SPM data	all	.gwy, .gsf
corrected images	all	.png, .svg
drift velocity	all	.csv
corrected lattice parameters	periodic	.csv
calibration parameters^a^	periodic	.csv
user input parameters	all	.csv

^a^Requires the user to input reference data for the expected surface geometry.

In addition to the corrected SPM data, drift velocities and lattice parameters (only for images with periodic structures) calculated during drift correction are available as a session report in csv format. Calibration parameters are also available for images with periodic structures in the same session report file if the user specified a reference surface. User input parameters specified during the evaluation are recorded by *un*Drift and can be downloaded in a separate csv file.

## Results and Discussion

To demonstrate the performance of *un*Drift, we will now apply the software to experimental situations where the drift correction is either especially challenging or time-consuming.

### Challenging experimental conditions

For the above presentation of the working principle of our software, we used AFM images with good atomic contrast and reasonable drift velocities. In experiments, however, it is not always possible to reach these desirable conditions, which is why we want to show that our software can also deal with challenging experimental conditions. We will demonstrate that *un*Drift can handle (1) drift velocities exceeding the slow scan rate, (2) images with only small usable parts, and (3) images exhibiting a weak contrast or low signal-to-noise ratio.

**Figure 4:**
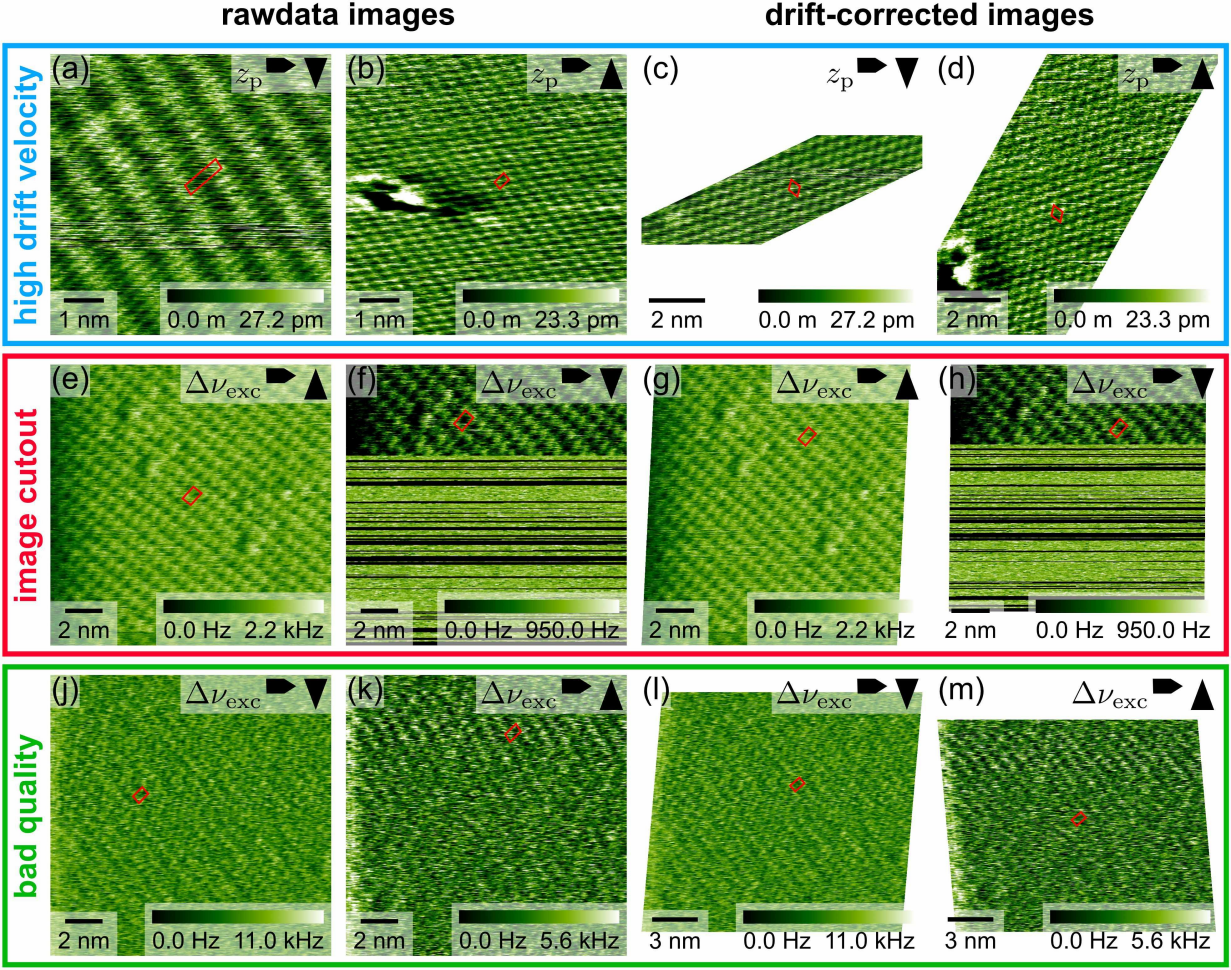
Example images demonstrating the applicability of *un*Drift to SPM experiments with high drift velocities (a–d), only partly usable images (e–h), and bad image quality (j–m). Raw-data images are shown on the left side of the figure, and the corresponding drift-corrected images are shown on the right side. In all images, the unit cell used for drift correction is shown as a red quadrangle. Images (a–d) show the atomic structure of calcium fluoride (111) recorded with high-resolution AFM in ultrahigh vacuum. Images (e–h) and (j–m) were recorded with high-resolution AFM at the calcite (10.4)–water interface.

First, we discuss a scenario with very high drift velocity. In Figure 4a,b, we show two consecutive AFM images of a calcium fluoride (111) surface recorded under ultrahigh vacuum conditions. The periodic structures observed in these two raw-data images (see red unit cells in Figure 4a,b) show a striking difference compared to each other and compared to the expected hexagonal structure. This obvious difference in appearance is caused by the strong image distortion associated with an exceptionally high drift velocity. High drift velocities can be challenging for drift correction, because the strong image distortion makes it difficult to identify pairs of features or corresponding lattice points. With *un*Drift, however, the images in Figure 4a,b can be drift-corrected easily, as algorithm I works reliably even for very high drift velocities.

We present the drift-corrected images corresponding to the images in Figure 4a,b in Figure 4c,d, respectively. Note that we cut the drift-corrected images to fit the form factor of this figure, while still being able to see the atomic structure. Figure 4c,d shows that the unit cell dimensions in both up and down image are now almost identical, and the surface now appears to be hexagonal. We find lattice parameters of 3.8 × 10^−10^ m and 3.9 × 10^−10^ m with an angle of 114°, which is very close to a hexagonal surface structure. The lattice parameters also agree well with lattice parameters documented in literature (*a* = 3.86 × 10^−10^ m and γ = 120°; [49]). We ascribe the slight deviation from the reference values to the experimental error, which we expect to be higher because of the exceptionally high drift velocity. We want to highlight that this is an extreme example where the absolute drift velocity (3.8 × 10^−11^ m·s^−1^) exceeds the scan velocity in the slow scan direction (3.7 × 10^−11^ m·s^−1^). For most experiments, the drift velocity will be much lower, as SPM experiments are typically optimized for stable conditions with low drift velocity. Nevertheless, this example shows that *un*Drift is not limited to low-drift environments but is also capable to correct SPM data recorded with (very) high drift velocities.

Second, we turn to images where only parts of the input images can be used for drift correction. During SPM experiments, sudden tip changes or collisions with the surface can significantly worsen or even destroy the observed contrast. Hence, it is quite common that only parts of an image can be used for evaluation. To demonstrate that *un*Drift can drift-correct these images by only considering (small) parts of an image, we show example images recorded at the calcite–water interface in Figure 4e,f. In our example, the sample drifted out of the scanner’s *z* range after the first third of the second image (Figure 4f), and the image contrast was completely lost. For drift correction, we used algorithm I based on the autocorrelation image and selected the usable part in Figure 4f manually, while the image in Figure 4e was used in its entirety. We show the drift-corrected results for both input images in Figure 4g,h, respectively. Comparison between the raw-data images (Figure 4e,f) and drift-corrected images (Figure 4g,h) shows that the unit cells of both images agree much better after drift correction. We conclude that the drift correction was successful, even though we could only use a small part of the second image for drift correction. This conclusion is confirmed by the derived unit cell dimensions of 5.1 × 10^−10^ m × 8.2 × 10^−10^ m with an angle of 89.8°, which fit the expected values (4.99 × 10^−10^ m × 8.10 × 10^−10^ m, 90.0°; [50]) within the experimental accuracy of our AFM instrument (distance accuracy: ±0.3 × 10^−10^ m, angle accuracy: ±2°; see below).

Third, we discuss the arguably most challenging situation for drift correction, images with an overall weak contrast and low signal-to-noise ratio. The periodic structures of the calcite–water interface in Figure 4j,k are very faint and, thus, hard to recognize by eye. Based on the autocorrelation function, however, *un*Drift can extract unit cell dimensions even from images with such weak contrast. The different dimensions of the unit cells extracted here (see red quadrangles in Figure 4j,k) reveal that these raw-data images are distorted by drift. We drift-corrected the raw-data images with algorithm I based on the autocorrelation image to obtain the drift-corrected images shown in Figure 4l,m. In the drift-corrected images, the periodic structures are still very faint, but the unit cell dimensions are now identical in both images. We derived lattice parameters of 5.0 × 10^−10^ m, 8.1 × 10^−10^ m, and 90.8°, which agree perfectly with the expected surface structure (4.99 × 10^−10^ m × 8.10 × 10^−10^ m, 90.0°; [50]). Hence, we conclude that *un*Drift also performs very well in the drift correction of images with weak contrast.

### Long image series

**Figure 5:**
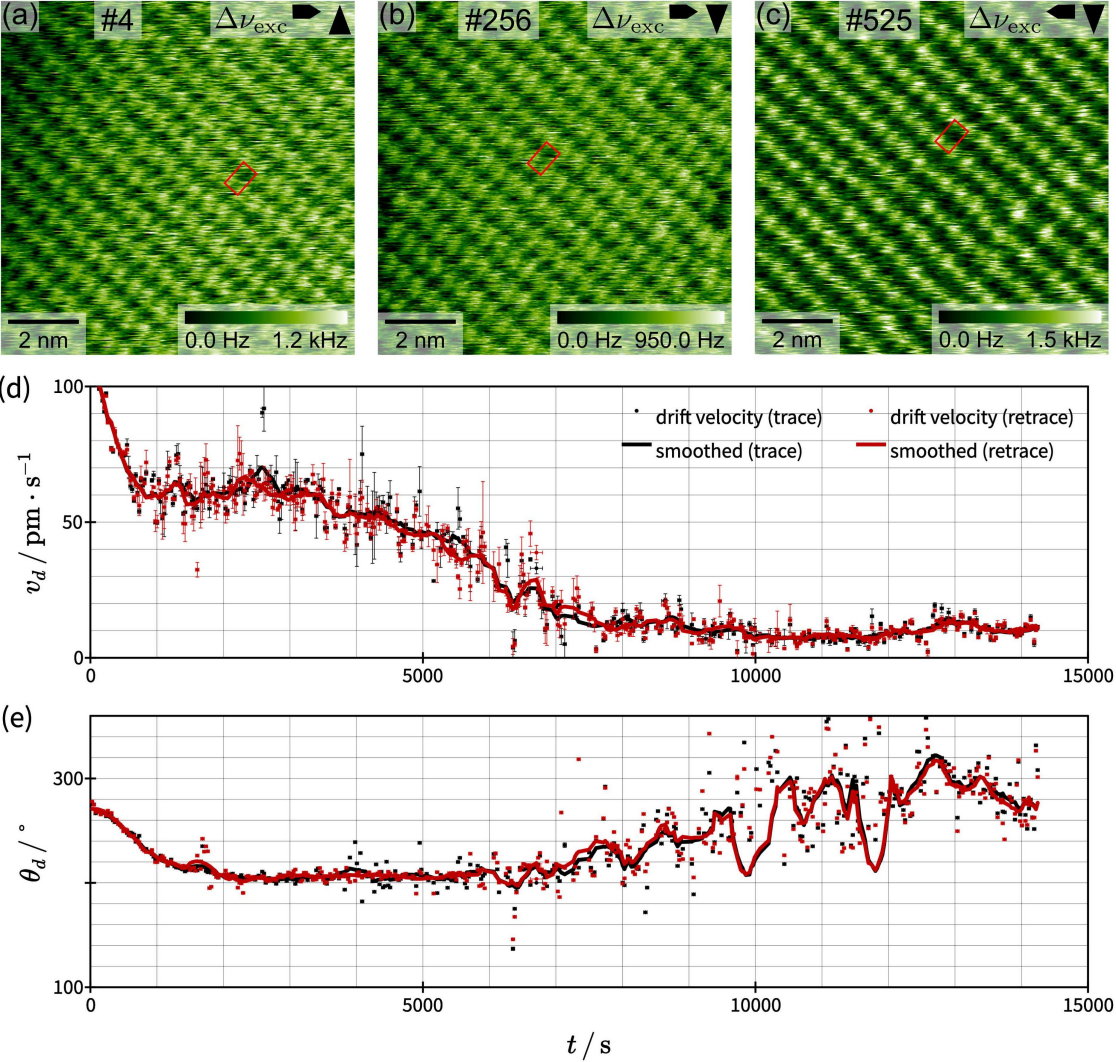
Application of *un*Drift to the analysis of a measurement session containing 530 AFM images recorded at the calcite–water interface. (a–c) Typical drift-corrected images from beginning, middle, and end of the evaluated AFM session. (d, e) Calculated drift velocities in polar coordinates as function of the measurement time *t*. The absolute values of 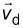 and the corresponding angle θ_d_ in are shown in (d) and (e), respectively. Results from trace and retrace images are shown as black and red circles, respectively, and the smoothed averages for both cases are shown as lines.

After discussing the performance of *un*Drift under different experimental conditions, we now demonstrate the applicability to long image series spanning several hundred SPM images with an example shown in Figure 5. The presented series comprises 530 high-resolution AFM images recorded at the calcite–water interface over a measurement time of approximately 4 h. We evaluated these images with the semi-automatic periodic analysis of *un*Drift (algorithm I) in approximately 1 h 40 min, which corresponds to a drift correction rate of 318 images per hour. In this series, the image contrast is rather stable over the full measurement time (see Figure 5a–c), facilitating drift correction. Thus, the given correction rate is likely to be the upper limit. We find that the number of drift-corrected images per hour and, consequently, the performance of *un*Drift strongly depend on the image quality and contrast stability. Moreover, it is typically faster to evaluate longer image series than several series with few images. We find that the average drift correction rate with *un*Drift for typical AFM images is between 100 images per hour for short series and 200 images per hour for long series. Note that the analysis times discussed here are limited by the manual steps of the analysis (i.e., adjusting the analysis parameters and checking the results) rather than the computation time on all tested common desktop computers (e.g., Windows 10, Intel Core i5-9500, 6 cores @ 3.00 GHz, 8.00 GB RAM).

Next, we discuss the drift velocities measured during this image series and what they tell us about our AFM instrument. In Figure 5d,e, we show the absolute values of *v*_d_ and polar angle θ_d_ of the drift velocity 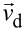 as functions of the time. We observe a steep decrease of the drift velocity during the first 800 s (15 min) of our experiment. Then, the drift velocity decreases further, but with a lower change rate, until it reaches stable conditions approximately 8000 s (2 h 15 min) after starting the experiment. The polar angle θ_d_ (see Figure 5e) follows the same trend for the first 6000 s. After that, the change in the scan angle increases again. Compared to the change at the beginning of the experiment (before 800 s), however, the change in scan angle after 6000 s is much more random and looks more like noise. We explain this observation with the decreasing absolute value of the drift velocity. At the beginning of the experiment, the drift is caused by equilibration processes between sample surface, liquid, and AFM instrument, so the absolute drift velocity is high and has a stable direction. Then, with increasing measurement time, the system gets closer to equilibrium, which causes the drift velocity to decrease. Hence, the relative contribution of thermal fluctuations to the drift velocity increases and the direction of the drift velocity becomes less stable. The trends observed in Figure 5d,e show us that our AFM instrument needs to equilibrate for about 800 s (15 min) to reach a moderate drift velocity. Stable measurement conditions, however, are only reached after approximately 8000 s (2 h 15 min) of equilibration time.

**Figure 6:**
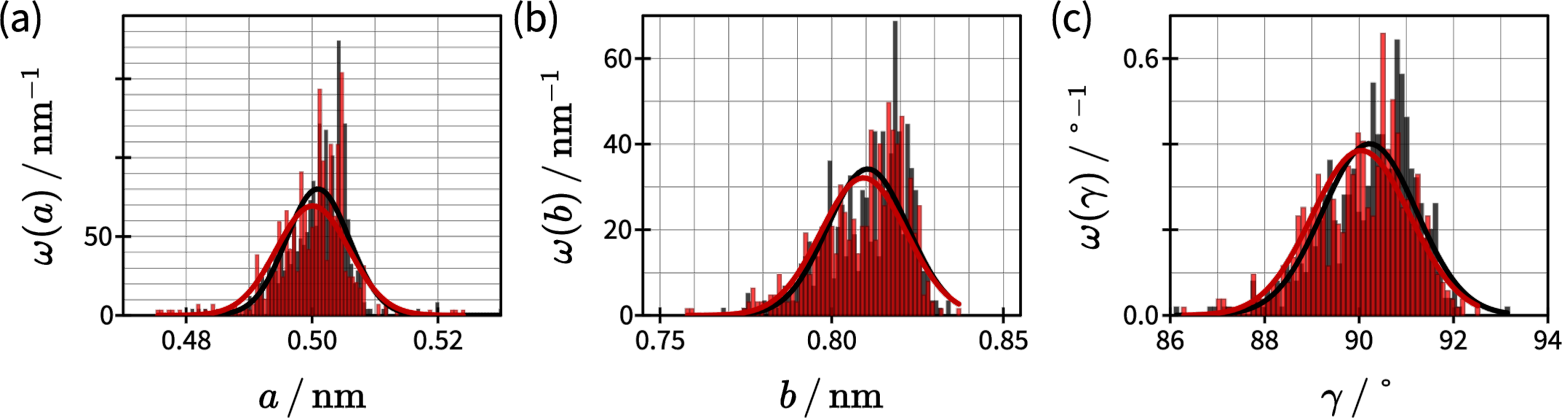
Histograms of lattice parameters *a*, *b*, and γ derived for the water structure at the calcite(10.4)–water interface based on the image series shown in Figure 5. Experimental results are shown as bar plots, and the corresponding normal distributions are shown as lines. Results for the trace (retrace) images are black (red). The average lattice parameters determined from these distributions are *a* = 5.0 × 10^−10^ m, *b* = 8.1 × 10^−10^ m, and γ = 90° for both trace and retrace data. The corresponding standard deviations are σ*_a_* = 0.06 × 10^−10^ m, σ*_b_* = 0.12 × 10^−10^ m, and σ_γ_ = 1.0°.

In addition to the drift velocities, we want to discuss the lattice parameters derived by *un*Drift. Figure 6 shows histograms for all three lattice parameters derived from all 530 images in the session shown in Figure 5. Because of the applied drift correction scheme, we get two sets of lattice parameters for each image. One dataset originates from drift correction with the previous image, and the other dataset originates from drift correction with the next image. Here, we show the histograms of the average values calculated for each image. Figure 6 reveals that all three experimental lattice parameters (5.0 × 10^−10^ m × 8.1 × 10^−10^ m, 90°) agree with the literature values (4.99 × 10^−10^ m × 8.10 × 10^−10^ m, 90.0°; [50]) within an interval of the standard deviation (σ*_a_* = 0.06 × 10^−10^ m, σ*_b_* = 0.12 × 10^−10^ m, and σ_γ_ = 1.0°) from the average value. Moreover, these histograms give us detailed insight into the experimental accuracy of our AFM measurements. Figure 6a,b reveals that our instrument’s distance accuracy after calibration and drift correction is ±2.5 × 10^−11^ m. In terms of angles, Figure 6c shows that the accuracy is ±2°. In both cases, we used the 2σ ≈ 0.95% confidence interval to determine the accuracy of our device. Note that the measurement accuracy also depends on the image quality, as the peaks in the autocorrelation and Fourier transform images get less defined with decreasing image quality. Hence, we expect the accuracy of distances and angles to decrease with decreasing image quality.

## Conclusion

We present *un*Drift, a versatile and powerful software for calibration and drift correction of SPM images. *un*Drift provides three different drift correction algorithms enabling the drift correction of SPM images, regardless of their scan direction and whether they exhibit periodic structures or not. We demonstrate the performance of *un*Drift in terms of image quality and experimental conditions with three examples, namely one image set with exceptionally high drift velocity, one with only a small usable part of an image, and one with an overall low image contrast and low signal-to-noise ratio. *un*Drift handles these three situations reliably. Moreover, we show that *un*Drift can be used for the fast evaluation of drift velocities and lattice parameters in long measurement sessions spanning several hundred images. We, thus, conclude that *un*Drift can be a valuable tool in the evaluation of SPM image data.

## Supporting Information

Supporting Information features derivations of the mathematical expressions used for the calibration and drift correction, a description of the procedure used to derive correction parameters as well as other technical aspects. Moreover, the gwy files for all shown AFM images and used analysis parameters are included in Supporting Information Files 2–5.

File 1 Calibration and drift correction procedures

File 2 Experimental data shown in Figure 2.

File 3 Experimental data shown in Figure 3.

File 4 Experimental data shown in Figure 4.

File 5 Experimental data shown in Figure 5.

## References

[1] Libioulle L, Ronda A, Taborelli M, Gilles J M (1991). J Vac Sci Technol, B: Microelectron Nanometer Struct–Process, Meas, Phenom.

[2] van de Leemput L E C, Rongen P H H, Timmerman B H, van Kempen H (1991). Rev Sci Instrum.

[3] Yurov V Y, Klimov A N (1994). Rev Sci Instrum.

[4] Yothers M P, Browder A E, Bumm L A (2017). Rev Sci Instrum.

[5] Rahe P, Bechstein R, Kühnle A (2010). J Vac Sci Technol, B: Microelectron Nanometer Struct–Process, Meas, Phenom.

[6] Henriksen K, Stipp S L S (2002). Am Mineral.

[7] Adler J G, Chen T T, Gallagher M C, Konkin M K, Mullin D P (1991). J Vac Sci Technol, B: Microelectron Nanometer Struct–Process, Meas, Phenom.

[8] Cai C Z, Chen X Y, Shu Q Q, Zheng X L (1992). Rev Sci Instrum.

[9] Carrara S, Facci P, Nicolini C (1994). Rev Sci Instrum.

[10] Stoll E P (1994). Rev Sci Instrum.

[11] Trawick M L, Megens M, Angelescu D E, Harrison C, Vega D A, Chaikin P M, Register R A, Adamson D H (2003). Scanning.

[12] Lapshin R V (2007). Meas Sci Technol.

[13] Follin N D, Taylor K D, Musalo C J, Trawick M L (2012). Rev Sci Instrum.

[14] Jørgensen J F, Carneiro K, Madsen L L, Conradsen K (1994). J Vac Sci Technol, B: Microelectron Nanometer Struct–Process, Meas, Phenom.

[15] Fu J, Chu W, Dixson R, Orji G, Vorburger T, Secula E M, Seiler D G, Khosla R P, Herr D, Garner C M (2009). AIP Conf Proc.

[16] Zhang L, Long Q, Liu Y, Zhang J, Feng Z (2016). Ultramicroscopy.

[17] Zhang L, Chen X, Huang J, Li H, Chen L, Huang Q (2019). Rev Sci Instrum.

[18] Jørgensen J F, Madsen L L, Garnaes J, Carneiro K, Schaumburg K (1994). J Vac Sci Technol, B: Microelectron Nanometer Struct–Process, Meas, Phenom.

[19] Staub R, Alliata D, Nicolini C (1995). Rev Sci Instrum.

[20] Poirier G E, White J M (1990). Rev Sci Instrum.

[21] Korpelainen V, Lassila A (2007). Meas Sci Technol.

[22] Marinello F, Savio E (2007). Meas Sci Technol.

[23] Rost M J, Crama L, Schakel P, van Tol E, van Velzen-Williams G B E M, Overgauw C F, ter Horst H, Dekker H, Okhuijsen B, Seynen M (2005). Rev Sci Instrum.

[24] Patera L L, Bianchini F, Africh C, Dri C, Soldano G, Mariscal M M, Peressi M, Comelli G (2018). Science.

[25] Diao Z, Ueda K, Hou L, Yamashita H, Custance O, Abe M (2023). Appl Phys Lett.

[26] Gómez-Rodríguez J M, Sáenz J J, Baró A M, Veuillen J-Y, Cinti R C (1996). Phys Rev Lett.

[27] Huang W, Wang W, Xia A, Jin N, Hu Z (2000). J Vac Sci Technol, B: Microelectron Nanometer Struct–Process, Meas, Phenom.

[28] Rahe P, Schütte J, Schniederberend W, Reichling M, Abe M, Sugimoto Y, Kühnle A (2011). Rev Sci Instrum.

[29] Clifford C A, Seah M P (2009). Meas Sci Technol.

[30] Niu D, Li J, Chen Y, Huang W (2010). J Vac Sci Technol, B: Microelectron Nanometer Struct–Process, Meas, Phenom.

[31] Gaponenko I, Tückmantel P, Ziegler B, Rapin G, Chhikara M, Paruch P (2017). Sci Rep.

[32] Luo T, Chen Y, Huang W, Gao S (2014). Proc SPIE.

[33] Sollböhmer O, May K-P, Anders M (1995). Thin Solid Films.

[34] Mantooth B A, Donhauser Z J, Kelly K F, Weiss P S (2002). Rev Sci Instrum.

[35] Chen Y, Huang W (2007). Rev Sci Instrum.

[36] Marinello F, Balcon M, Schiavuta P, Carmignato S, Savio E (2011). Meas Sci Technol.

[37] Woodward J T, Schwartz D K (1998). J Vac Sci Technol, B: Microelectron Nanometer Struct–Process, Meas, Phenom.

[38] Sun Y, Pang J H L (2006). Nanotechnology.

[39] Marinello F, Bariani P, Chiffre L D, Savio E (2007). Meas Sci Technol.

[40] Salmons B S, Katz D R, Trawick M L (2010). Ultramicroscopy.

[41] Degenhardt J, Tutsch R, Dai G (2021). Meas Sci Technol.

[42] Kizu R, Misumi I, Hirai A, Gonda S (2020). Meas Sci Technol.

[43] Meyer T R, Ziegler D, Brune C, Chen A, Farnham R, Huynh N, Chang J-M, Bertozzi A L, Ashby P D (2014). Ultramicroscopy.

[44] Sun X, Heaps E, Yacoot A, Yang Q, Grolich P, Klapetek P (2021). Meas Sci Technol.

[45] Horcas I, Fernández R, Gómez-Rodríguez J M, Colchero J, Gómez-Herrero J, Baro A M (2007). Rev Sci Instrum.

[46] (2023). unDrift - SPM Image Drift Correction and Calibration.

[47] (2021). Kontrast: interactive data visualization.

[48] Nečas D, Klapetek P (2012). Cent Eur J Phys.

[49] Deer W A, Howie R A, Zussman J (1992). An introduction to the rockforming minerals.

[50] Effenberger H, Mereiter K, Zemann J (1981). Z Kristallogr.

