# Supplementary material for: unDrift: A versatile software for fast offline SPM image drift correction
Source: Beilstein J Nanotechnol. 2023 Dec 28;14:1225–37. doi: 10.3762/bjnano.14.101 (PMC10760460; doi:10.3762/bjnano.14.101)
Supplement: File 1 — Calibration and drift correction procedures [file Beilstein_J_Nanotechnol-14-1225-s001.pdf]

BEILSTEIN JOURNAL OF NANOTECHNOLOGY

Supporting Information

for

*un*Drift: A versatile software for fast offline SPM image drift correction

Tobias Dickbreder, Franziska Sabath, Lukas Höltkemeier, Ralf Bechstein and Angelika Kühnle

Beilstein J. Nanotechnol. **2023,** *14,* 1225–1237. doi:10.3762/bjnano.14.101

Calibration and drift correction procedures

License and Terms: This is a supporting information file under the terms of the Creative Commons Attribution License (https://creativecommons.org/licenses/by/4.0). Please note that the reuse, redistribution and reproduction in particular requires that the author(s) and source are credited and that individual graphics may be subject to special legal provisions.
The license is subject to the *Beilstein Journal of Nanotechnology* terms and conditions: (https://www.beilstein-journals.org/bjnano/terms)

Contents

1 Lateral Calibration **S3**

1.1 Physical Background S3

1.2 Coordinate Systems S4

1.2.1 Scanner Coordinates S5

1.2.2 Microscope Coordinates S6

1.2.3 Image Coordinates S7

1.3 Coordinate Transformations S9

1.4 Determination of Calibration Parameters S10

1.4.1 General Procedure S11

1.4.2 Implementation in *un*Drift S14

2 Drift Correction **S15**

2.1 Inverse Drift Transformation S15

2.2 Time Mapping S19

2.2.1 Regular Scanning S21

2.2.2 Over-scanning S22

2.3 Determination of the Drift Velocity S23

2.3.1 Distortion of the Surface Periodicity S23

2.3.2 Stationary Features and Structures S25

3 Experimental Methods **S28**

3.1 AFM in Ultrahigh Vacuum S28

3.2 AFM at the Solid–Liquid Interface S29

3.3 Legend in AFM Images S29

4 Additional Experimental Data **S30**

5 Author Contributions **S30**

The physical and mathematical models behind scanning probe microscopy (SPM) image calibration [1-13] and drift correction [12-30] are well known in literature. However, as drift correction and calibration strategies have been (and still are) developed by many authors over several decades, the mathematical description and wording are not always consistent between different sources. To clarify the models we use in *un*Drift, we decided to summarize our understanding of calibration and drift correction in the following Supporting Information.

1 Lateral Calibration

1.1 Physical Background

In scanning probe microscopy, images of the sample are obtained by moving the probe and recording the probe–sample interaction at fixed points covering the area or volume of interest. Typically, this movement is realized with three piezo-electric actuators that translate an input voltage V_i into a spacial displacement in one of the three spacial directions $i = x, y, z$ [1,31]. The alignment of these piezo-actuators, however, is never perfect, which is why the piezo actuators often cause some movement in the other two spacial directions as well [2-4]. In general, the displacement $\vec{r}_i$ is a complex, non-linear functional of the input voltage V_i [9-11,32]. This non-linear dependence of the displacement on the voltage is the origin of measurement artifacts such as creep [8-10,24,32] and piezo hysteresis [7,9-11,32-34].

Here, we only consider linear piezo movement as this is, especially for atomic-scale images, the decisive contribution to piezo movement [11]. In the case of linear piezo movement, the applied voltage V_l is connected to the spacial displacement by Equation (S1), where c_l is the constant linear calibration factor and $\vec{e}_{\mathrm{p},l}$ the unit vector of piezo movement in l direction. The applied voltage V_l and calibration factor c_l can be combined to the piezo displacement $l_\mathrm{p} = c_l V_l$.

$$\vec{r}_l = c_l V_l \vec{e}_{\mathrm{p},l} = l_\mathrm{p} \vec{e}_{\mathrm{p},l} \tag{S1}$$

For a quantitative analysis of SPM data, it is crucial that the piezo actuators of the used scanning probe microscope are calibrated precisely. Otherwise, distances and angles extracted from SPM images will be incorrect [2-4,11]. The calibration of piezo actuators, however, can change depending on the measurement temperature or due to piezo aging [13,31]. Thus, it is important to check the instrument's calibration regularly. Also, it becomes interesting to correct SPM images with an imperfect calibration after the measurement. In this chapter, we will discuss both aspects, as *un*Drift provides features to check an existing calibration and handle SPM data with an imperfect calibration. We will not discuss the (initial) calibration of SPM instruments as the corresponding procedures and, especially, the calibration parameters depend on the specific instrument and control software.

1.2 Coordinate Systems

To discuss the lateral calibration of scanning probe microscopes, we consider three coordinate systems, we refer to as (a) the scanner coordinate system, (b) the microscope coordinate system, and (c) the image coordinate system (see Figure S1a–c and the first three entries in Table S1). The scanner coordinate system represents the real, physical world, while the microscope and image coordinate systems reflect the current calibration of the SPM instrument. As data from SPM experiments is processed by the SPM instrument, all SPM data is initially stored in the microscope or image coordinate system. Note that literature [2-4,11,32] typically only discusses two coordinate systems, that is, for the not calibrated (image coordinates) and calibrated (scanner coordinates) data. However, in these considerations [2-4,11,32] the effect of different scan angles on the calibration is hidden within the absolute orientation of the scanner coordinate system. Here, we discuss a third coordinate system, the microscope coordinates, to show how the calibration depends on the scan angle.

For a perfect calibration, all three coordinate systems discussed here are equivalent [2-4,11]. In reality, however, we often have an imperfect calibration, which leads to a deviation between the real world (scanner coordinates) and the SPM data (microscope or image coordinates). This means that the basis vectors of the microscope and image coordinates are not orthonormal anymore (see Figure S1d), which typically leads to incorrect distances and angles [2-4,11]. To correct artifacts of imperfect

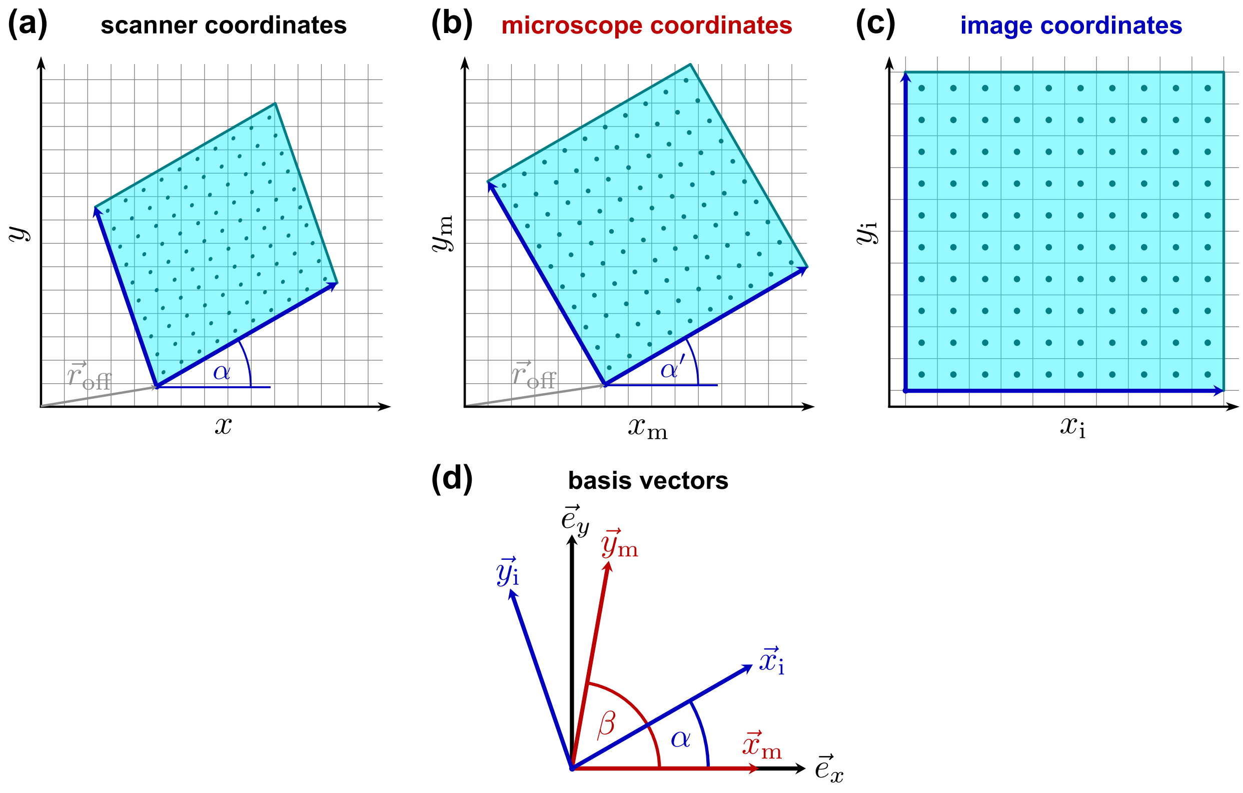

Figure S1: Schematic representation of the three coordinate systems relevant for lateral SPM calibration: (a) scanner coordinate system (b) microscope coordinate system (c) image coordinate system. In (a–c) an example scan area is shown by a cyan parallelogram and the positions of the raster points are indicated by cyan circles. The scan vectors in the fast and the slow scan direction are shown as dark blue arrows. (d) Basis vectors of all three coordinate systems in the scanner coordinate system.

calibration in SPM data, we first discuss all three coordinate systems and the relations between their basis vectors. Then, we derive transformations from the microscope and image coordinates into scanner coordinates.

1.2.1 Scanner Coordinates

First, we construct the scanner coordinate system as an orthonormal coordinate system with correct physical measures for distances and angles. To this end, we can use the standard canonical basis vectors $\vec{e}_x$ and $\vec{e}_y$ as the basis vectors in x and y directions. The corresponding vector components are x and y, respectively. As the (arbitrary) origin of the scanner coordinate system, we choose the resting position of the scanner, that is, the scanner position when no voltages are applied to the piezo

Table S1: Summary of all four coordinate systems used to describe lateral calibration and drift correction of SPM data. The first three coordinate systems, that is, scanner, microscope, and image coordinate systems, are used for the instrument calibration. The fourth coordinate system, the surface coordinate system, is needed to describe drift.

coordinates	basis	components	origin	description
scanner	$\{\vec{e}_x, \vec{e}_y\}$	(x, y)	$\vec{0}$	orthonormal coordinate system used to describe the real, physical movement of the scanner
microscope	$\{\vec{x}_\mathrm{m}, \vec{y}_\mathrm{m}\}$	$(x_\mathrm{m}, y_\mathrm{m})$	$\vec{0}$	coordinate system used by the SPM instrument to control the scanner movement
image	$\{\vec{x}_\mathrm{i}, \vec{y}_\mathrm{i}\}$	$(x_\mathrm{i}, y_\mathrm{i})$	$\vec{r}_\mathrm{off}$	coordinate system used for display and storage of SPM data
surface	$\{\vec{e}_x, \vec{e}_y\}$	$(x_\mathrm{surf}, y_\mathrm{surf})$	$-\vec{d}(t)$	orthonormal coordinate system fixed at one point on the surface

actuators. After fixing the origin position, the scanner coordinate system has one remaining degree of freedom, namely the total rotation. Without loss of generality, we can choose any given rotation, as this does not affect the surface structure observed in the images. In our case, it is advantageous to choose the $\vec{e}_x$ vector parallel to the x direction of the SPM instrument. Given that the SPM instrument applies only a voltage at the x piezo to achieve the motion in x direction, this means that the $\vec{e}_x$ vector is also parallel to the x piezo displacement $\vec{e}_{\mathrm{p},x}$. Otherwise, the $\vec{e}_x$ vector points in a direction chosen by the instruments calibration. In both cases, the absolute orientation is not known and needs to be measured if relevant for the experiments.

1.2.2 Microscope Coordinates

Second, we discuss the microscope coordinates. The microscope coordinate system is used by the SPM instrument during the measurement to calculate the scanner position from the input voltages and to control the measurement path. In order to do so, the SPM instrument assumes that the basis vectors of the microscope coordinate system $\vec{x}_\mathrm{m}$ and $\vec{y}_\mathrm{m}$ (components x_m and y_m) are orthogonal and normalized. However, this is only the case for a perfect calibration. In case of an imperfect calibration, the microscope coordinate system is not orthonormal and, thus, the intended and real

scanner movements deviate. As a consequence, the recorded SPM data in microscope coordinates is distorted.

The first basis vector of the microscope coordinates, $\vec{x}_{\mathrm{m}}$, is parallel to the x direction of the SPM instrument. As we already defined that $\vec{e}_x$ is parallel to the same direction, $\vec{x}_{\mathrm{m}}$ and $\vec{e}_x$ are parallel to each other. Additionally, we need to consider that the length of $\vec{x}_{\mathrm{m}}$ might not be unity in case of an imperfect calibration. Both of which is included in Equation (S2), where the dimensionless correction factor κ_x is the length of $\vec{x}_{\mathrm{m}}$.

$$\vec{x}_{\mathrm{m}} = \kappa_x \vec{e}_x \tag{S2}$$

The second basis vector of the microscope coordinate system is $\vec{y}_{\mathrm{m}}$. In general, $\vec{y}_{\mathrm{m}}$ can have any given length and direction; hence, it might be neither orthogonal to $\vec{x}_{\mathrm{m}}$ nor normalized. We describe the direction of $\vec{y}_{\mathrm{m}}$ by the angle β between $\vec{y}_{\mathrm{m}}$ and $\vec{x}_{\mathrm{m}}$, which is also the angle between $\vec{y}_{\mathrm{m}}$ and $\vec{e}_x$. The length of $\vec{y}_{\mathrm{m}}$ is — analogous to the description of $\vec{x}_{\mathrm{m}}$ — given by the correction factor κ_y. Combining both, we arrive at Equation (S3), where $\boldsymbol{R}_\beta$ is the rotation matrix for a rotation around β in scanner coordinates. (The rotation matrix in two dimensions is given by $\boldsymbol{R}_\beta = \begin{pmatrix} \cos\beta & -\sin\beta \\ \sin\beta & \cos\beta \end{pmatrix}$.)

$$\vec{y}_{\mathrm{m}} = \kappa_y \boldsymbol{R}_\beta \vec{e}_x \tag{S3}$$

The origin of the microscope coordinate system is located in the resting position of the piezo actuator, which means that the origins of the scanner and microscope coordinate systems coincide.

1.2.3 Image Coordinates

Third, we turn to the image coordinate system, which is typically used to store and display SPM images [2-4,11]. The image coordinate system is indeed very suitable for displaying and storing image data as the basis vectors in x and y directions are parallel to the fast and slow scan directions,

respectively. Hence, the recorded pixels are rectangular and align with the image coordinate system (see Figure S2 (c)), which is very advantageous for the storage and display of SPM data. We refer to the basis vectors of the image coordinates as $\vec{x}_\mathrm{i}$ and $\vec{y}_\mathrm{i}$, and the corresponding vector components are x_i and y_i.

Compared to the microscope coordinates, the image coordinate system deviates in its origin and rotation. As the origin of the image coordinates, we use the bottom left corner of the image as opposed to the resting position of the scanner in microscope coordinates. Moreover, the image coordinates are rotated relative to the microscope coordinates by the apparent scan angle α', which creates the discussed alignment between basis vectors and scan directions. Here, the apparent scan angle α' is the intended scan angle chosen during the measurement (see Figure S2b). Note that, depending on the quality of the calibration, α' might not coincide with the real scan angle α of the scanner motion (see Figure S2a; in general, the apparent and the real scan angle only coincide if the calibration is either perfect or $\kappa_x = \kappa_y$ and $\beta = 90°$.).

We can derive the basis vectors of the image coordinates $\vec{x}_\mathrm{i}$ and $\vec{y}_\mathrm{i}$ by rotation of the microscope basis with a rotation matrix $\boldsymbol{R}'_{\alpha'}$. It is important to note that the image coordinates are originally defined by the SPM instrument during the measurement; thus, they are defined relative to the microscope coordinates. As a consequence, we need to apply the rotation matrix $\boldsymbol{R}'_{\alpha'}$ in microscope coordinates as well. It only affects the x_m and y_m components of a vector in microscope coordinates and not the corresponding basis vectors. Following this derivation, we arrive at Equations (S4) and (S5) for the basis vectors of the image coordinates.

$$\vec{x}_\mathrm{i} = \boldsymbol{R}'_{\alpha'}\vec{x}_\mathrm{m} = \cos\alpha'\vec{x}_\mathrm{m} + \sin\alpha'\vec{y}_\mathrm{m} \tag{S4}$$

$$\vec{y}_\mathrm{i} = \boldsymbol{R}'_{\alpha'}\vec{y}_\mathrm{m} = -\sin\alpha'\vec{x}_\mathrm{m} + \cos\alpha'\vec{y}_\mathrm{m} \tag{S5}$$

1.3 Coordinate Transformations

In order to correct the effect of an imperfect calibration on SPM data, we need to transform the raw data from non-orthonormal image or microscope coordinates into orthonormal scanner coordinates [2-4,11]. These transformations are performed with the transformation matrices that we derive in this section. Note that we need both transformations from image coordinates into scanner coordinates and from microscope coordinates into scanner coordinates, as the image data is typically stored in image coordinates and the image offset is stored in microscope coordinates.

The transformation from image coordinates into scanner coordinates is an affine transformation given by Equation (S6), where $\boldsymbol{K}$ is the corresponding transformation matrix [1]. Here, $\vec{r}_{\text{off}}$ is the location vector of the bottom left corner of the scan area, that is, the origin of the image coordinates, in scanner coordinates as shown in Figure S1a.

$$\vec{r} = \boldsymbol{K} \begin{pmatrix} x_{\text{i}} \\ y_{\text{i}} \end{pmatrix} + \vec{r}_{\text{off}} \tag{S6}$$

According to linear algebra, the elements of the transformation matrix $\boldsymbol{K}$ are given by the scalar products between the basis vectors of both coordinate systems according to Equation (S7). For the evaluation of the scalar products in Equation (S7), it is crucial to use the vector representations in scanner coordinates, because the standard scalar product only applies in orthonormal coordinates. ("Standard scalar product" refers to $\vec{a} * \vec{c} = a_1 c_1 + a_2 c_2$. This scalar product is limited to orthonormal coordinates as the basis vectors need to be considered explicitly for non-orthonormal coordinates. In that case, the scalar product would be given by $\vec{a} * \vec{c} = \sum_{i=1}^{2} \sum_{k=1}^{2} a_i c_k \vec{b}_i * \vec{b}_k$, where $\vec{b}_i$ are the basis vectors of the non-orthonormal vector space.) Otherwise, the effect of non-orthogonality needs to be included in the scalar product.

$$\boldsymbol{K} = \begin{pmatrix} \vec{e}_x * \vec{x}_\mathrm{i} & \vec{e}_x * \vec{y}_\mathrm{i} \\ \vec{e}_y * \vec{x}_\mathrm{i} & \vec{e}_y * \vec{y}_\mathrm{i} \end{pmatrix} = \begin{pmatrix} \kappa_x \cos\alpha' + \kappa_y \sin\alpha' \cos\beta & -\kappa_x \sin\alpha' + \kappa_y \cos\alpha' \cos\beta \\ \kappa_y \sin\alpha' \sin\beta & \kappa_y \cos\alpha' \sin\beta \end{pmatrix} \tag{S7}$$

Next, we discuss the transformation from microscope coordinates into scanner coordinates. As the origins of the microscope and scanner coordinates coincide, the transformation from microscope coordinates to scanner coordinates is a linear transformation according to Equation (S8). Here, $\boldsymbol{K}'$ is again the corresponding transformation matrix.

$$\vec{r} = \boldsymbol{K}' \begin{pmatrix} x_\mathrm{m} \\ y_\mathrm{m} \end{pmatrix} \tag{S8}$$

The derivation of $\boldsymbol{K}'$ is analogous to the derivation of $\boldsymbol{K}$. Thus, we arrive at Equation (S9) for the transformation matrix from microscope into scanner coordinates.

$$\boldsymbol{K}' = \begin{pmatrix} \vec{e}_x * \vec{x}_\mathrm{m} & \vec{e}_x * \vec{y}_\mathrm{m} \\ \vec{e}_y * \vec{x}_\mathrm{m} & \vec{e}_y * \vec{y}_\mathrm{m} \end{pmatrix} = \begin{pmatrix} \kappa_x & \kappa_y \cos\beta \\ 0 & \kappa_y \sin\beta \end{pmatrix} \tag{S9}$$

1.4 Determination of Calibration Parameters

A variety of different methods is documented for the determination of calibration parameters for scanning probe microscopes [2-6,10-13,31]. One of the available techniques is the use of calibration gratings [5,6,10,31], that is, special surface structures prepared to exhibit a well-defined periodic ridge structure in one or two dimensions. However, these gratings are only available on the scales of micrometers and several hundred nanometers, which is why they are not suitable for a calibration on the atomic scale. Instead, we need a calibration standard on the atomic length scale to calibrate our devices and assess the quality of our calibration. Atomic-scale calibrations are typically carried

out based on the periodic structure of known crystal surfaces such as highly oriented pyrolytic graphite (HOPG) [2-4,10-13]. In our experiments, we use the calcite(10.4) surface as an atomic-scale calibration standard. Note that an atomic-scale calibration is necessary to achieve reliable results, as the sensitivity of piezoelectric actuators can vary considerably depending on the length scale [11].

1.4.1 General Procedure

In order to use the surface structure of crystalline samples as an atomic-scale calibration standard, we need a method to extract calibration parameters from the apparent lattice vectors observed in SPM images. These calibration parameters could be derived in a straightforward manner by solving a simple linear equation system, if we knew the real lattice vectors of the sample surface $\vec{a}$ and $\vec{b}$ [2-4]. Unfortunately, the real lattice vectors are hard to quantify with the necessary precision, as they include the absolute orientation of the sample, which is typically only known to within $\pm 1°$. For this reason, we use a method that does not include the absolute orientation but only the lattice parameters a, b, and γ of the surface used for calibration.

Next, we derive this calibration method, which only uses the observed lattice vectors in image coordinates ($\vec{a}'$ and $\vec{b}'$) and the lattice parameters of the real surface structure (a, b, and γ) as input parameters to calculate the calibration parameters κ_x, κ_y, and β. The scalar product of the real lattice vector $\vec{a}$ with itself is given by $\vec{a}^{\mathsf{T}}\vec{a} = a^2$. In this equation, we can replace the lattice vector $\vec{a}$ by the product of the corresponding image lattice vector $\vec{a}'$ with the calibration matrix $\boldsymbol{K}$ (see Equation (S7)). This replacement lets us arrive at Equation (S10), where $\boldsymbol{M} = \boldsymbol{K}^{\mathsf{T}}\boldsymbol{K}$ is an auxiliary matrix defined by Equation (S11). Here, it is important to note two facts about the matrix $\boldsymbol{M}$. First, the matrix $\boldsymbol{M}$ contains the dependence of Equation (S10) on the calibration matrix $\boldsymbol{K}$; hence, it also contains the searched calibration parameters κ_x, κ_y, and β. Second, $\boldsymbol{M}$ is identical with its transposed form $\boldsymbol{M}^{\mathsf{T}}$; hence, the matrix $\boldsymbol{M}$ only has three independent matrix elements m_{11}, m_{22} and $m_{12} = m_{21}$.

$$a^2 = \vec{a}^{\mathsf{T}}\vec{a} = \vec{a}'^{\mathsf{T}}\boldsymbol{M}\vec{a}' \tag{S10}$$

$$\boldsymbol{M} = \boldsymbol{K}^{\mathsf{T}}\boldsymbol{K} = \begin{pmatrix} k_{11}^2 + k_{21}^2 & k_{11}k_{12} + k_{21}k_{22} \\ k_{11}k_{12} + k_{21}k_{22} & k_{12}^2 + k_{22}^2 \end{pmatrix} \tag{S11}$$

The same considerations for the scalar products $\vec{b}^{\mathsf{T}}\vec{b}$ and $\vec{a}^{\mathsf{T}}\vec{b}$ yield Equations (S12) and (S13).

$$b^2 = \vec{b}^{\mathsf{T}}\vec{b} = \vec{b}'^{\mathsf{T}}\boldsymbol{M}\vec{b}' \tag{S12}$$

$$ab\cos\gamma = \vec{a}^{\mathsf{T}}\vec{b} = \vec{a}'^{\mathsf{T}}\boldsymbol{M}\vec{b}' \tag{S13}$$

With Equations (S10), (S12) and (S13), we have the three equations necessary to calculate all three matrix elements of our auxiliary matrix and, thus, to calculate the three calibration parameters. We can, now, write equations Equations (S10), (S12) and (S13) explicitly in terms of the elements of the image lattice vectors a'_1, a'_2, b'_1 and b'_2 and combine the results in the matrix Equation (S14). Equation (S14) is an ordinary linear equation system, which can be solved (numerically) for the auxiliary matrix elements with standard algorithms such as Gaussian elimination.

$$\begin{pmatrix} a^2 \\ b^2 \\ ab\cos\gamma \end{pmatrix} = \begin{pmatrix} a_1'^2 & 2a'_1a'_2 & a_2'^2 \\ b_1'^2 & 2b'_1b'_2 & b_2'^2 \\ a'_1b'_1 & a'_1b'_2 + a'_2b'_1 & a'_2b'_2 \end{pmatrix} \cdot \begin{pmatrix} m_{11} \\ m_{12} \\ m_{22} \end{pmatrix} \tag{S14}$$

After solving Equation (S14) for the matrix elements of $\boldsymbol{M}$, we can use the results to calculate the calibration parameters. To this end, the matrix elements m_{11}, m_{12}, and m_{22} as functions of the three calibration parameters κ_x, κ_y, and β are given by Equations (S7) and (S11). We first insert

Equation (S7) in Equation (S11) to achieve a system of three equations, which we then solve for the calibration parameters to arrive at Equations (S15) to (S17).

$$\kappa_x = \sqrt{\cos^2 \alpha' m_{11} - 2 \sin \alpha' \cos \alpha' m_{12} + \sin^2 \alpha' m_{22}} \tag{S15}$$

$$\kappa_y = \sqrt{\sin^2 \alpha' m_{11} + 2 \sin \alpha' \cos \alpha' m_{12} + \cos^2 \alpha' m_{22}} \tag{S16}$$

$$\cos \beta = \frac{1}{\kappa_x \kappa_y} \left(\sin \alpha' \cos \alpha' (m_{11} - m_{22}) + (\cos^2 \alpha' - \sin^2 \alpha') m_{12} \right) \tag{S17}$$

In summary, we propose a two-step process for the calculation of calibration parameters from atomic-scale SPM images. First, the elements of an auxiliary matrix $\boldsymbol{M}$ are calculated from the observed image lattice vectors by solving Equation (S14). Second, the actual calibration parameters κ_x, κ_y, and β are calculated from the auxiliary matrix elements with Equations (S15) to (S17), respectively. We can apply the same method if the observed lattice vectors $\vec{a}'$ and $\vec{b}'$ are already in microscope coordinates. In this case, the auxiliary matrix is given by $\boldsymbol{M}' = \boldsymbol{K}'^{\mathsf{T}} \boldsymbol{K}'$ where $\boldsymbol{K}'$ is the transformation matrix from microscope coordinates into scanner coordinates. The conditional Equation (S14) remains unchanged, except that the matrix elements of $\boldsymbol{M}$ are exchanged for those of $\boldsymbol{M}'$. The equations for the calibration parameters as functions of the auxiliary matrix elements (Equations (S18) to (S20)), however, become significantly simpler, as the effect of the coordinate system rotation associated with the (apparent) scan angle is removed from the equations. Naturally, this solution is identical with the solution found for the transformation from image coordinates into scanner coordinates with an apparent scan angle of $\alpha' = 0°$.

$$\kappa_x = \sqrt{m'_{11}} \tag{S18}$$

$$\kappa_y = \sqrt{m'_{22}} \tag{S19}$$

$$\cos\beta = \frac{m'_{12}}{\sqrt{m'_{11} m'_{22}}} \tag{S20}$$

1.4.2 Implementation in *un*Drift

For the technical implementation of this calibration procedure in *un*Drift, we need to consider two additional factors. Namely, we need to extract the lattice vectors from the input SPM data, and the apparent lattice parameters will not only be affected by a possibly imperfect calibration but also by drift [3,4,12]. To solve these problems, we arrive at a slightly more complicated procedure for the calculation of calibration parameters.

First, apparent lattice vectors are extracted from SPM images as described in the main text. As for the drift correction, we apply a semi-automatic procedure for the detection of lattice vectors based on Fourier transform or autocorrelation images. In the next step, these lattice vectors are drift-corrected in the microscope coordinate system without any calibration as discussed by Rahe et al. [20]. This yields apparent lattice vectors, which are drift-free and only contain the effects of a possibly imperfect calibration. Finally, we calculate calibration parameters from the lattice vectors and reference lattice parameters as described in the previous paragraphs.

2 Drift Correction

As discussed before, SPM images are not obtained by one concerted measurement, but by subsequent measurements at all pixel positions within the image. During this process, thermal changes can cause a movement of the scanner head relative to the sample surface, which adds to the tip movement caused by scanning [20,29]. This is commonly referred to as (thermal) drift. As a consequence of drift, surface structures observed in the image appear distorted, because the data recorded by the SPM only describes the tip position relative to the scanner head but not relative to the sample surface [20,29,32]. To obtain the real surface structure instead, we can either adjust the tip movement during the measurement to compensate for drift [28,30,35-37] (online drift correction) or correct the measured data for the additional movement between scanner head and sample surface afterwards [14-21,38-40] (offline drift correction). Here, we describe three simple procedures for offline drift correction and the underlying mathematical model.

2.1 Inverse Drift Transformation

To correct the effect of drift in SPM images, we need to find a suitable mathematical model for the observed image distortion. To this end, we consider two coordinate systems to describe the tip positions in the raw data and drift-corrected data sets. The raw data acquired by the SPM only accounts for the tip movement relative to the scanner, which is why we describe it with the scanner coordinate system discussed in section 1.2. For the drift-corrected data we are interested in the tip movement relative to the surface to ensure that surface features are displayed without distortion. Therefore, we introduce a new coordinate system for the drift-corrected data, which is fixed with respect to a point on the sample surface (surface coordinates, see Table S1). As the scanner and sample can move relative to each other during the measurement, the scanner and surface coordinates are shifted by a time-dependent offset $\vec{d}(t)$ as shown in Figure S2a. Consequently, the tip position in surface coordinates $\vec{r}_{\mathrm{surf}}(t)$ is related to the tip position in scanner coordinates $\vec{r}(t)$ by Equation (S21).

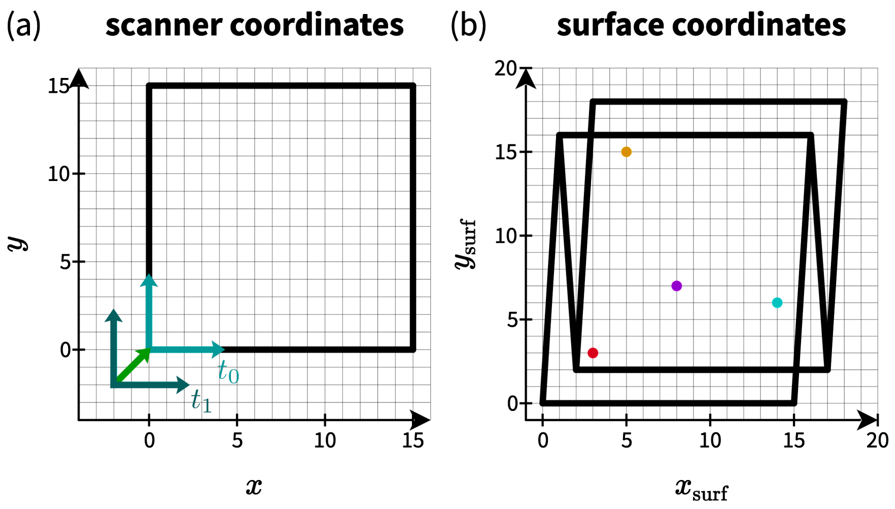

Figure S2: Schematic representation of the difference between scanner and surface coordinates. (a) Scan area of three consecutive images and origin of the surface coordinates in scanner coordinates. The origins of the surface coordinates for two different points in time t_0 and t_1 are shown as cyan coordinate crosses. At t_0, the scanner and surface coordinates coincide. The offset associated with t_1 is shown as a green vector. (b) Scan area of three consecutive images in surface coordinates. The positions of four stationary features on the surface are marked with colored dots. In both figure parts, the scan areas of the images are shown as black parallelograms. Note that all three scan areas coincide in scanner coordinates but not in surface coordinates.

$$\vec{r}_{\mathrm{surf}}(t) = \vec{r}(t) - \vec{d}(t) \tag{S21}$$

In this model, the time-dependencies of $\vec{r}_{\mathrm{surf}}(t)$ and $\vec{r}(t)$ describe the scanning motion in surface and scanner coordinates, respectively, while the relative motion between scanner and surface and, thus, the drift is solely contained within the time-dependence of the offset vector $\vec{d}$. The absolute value of $\vec{d}$, however, is arbitrary, as the origin of the surface coordinate system can be any point on the surface. To separate the effect of drift from this arbitrary offset, we introduce the drift velocity $\vec{v}_{\mathrm{d}}$ as the time derivative of $\vec{d}$ according to Equation (S22).

$$\vec{v}_{\mathrm{d}}(t) = \frac{\mathrm{d}}{\mathrm{d}t}\vec{d}(t) \tag{S22}$$

With this definition, the offset vector becomes the integral over the drift velocity as given in Equation (S23).

$$\vec{d}(t) = \int_0^t \vec{v}_\mathrm{d}(t)\mathrm{d}t \tag{S23}$$

So far, we did not make any assumptions on the functional form of offset vector or drift velocity as functions of time. Equations (S21) and (S23) can be used to correct SPM data for any known drift velocity, but the determination of such drift velocities of unknown time-dependence can be challenging. For many SPM experiments, however, the drift velocity only changes negligibly during the measurement of an image [13,20,28]. Therefore, we can simplify the drift correction considerably by assuming a constant drift velocity [13,20]. In this case, Equation (S23) simplifies to Equation (S24), where t is the time since the beginning of the image and $\vec{d}_0$ is the offset vector for $t = 0$.

$$\vec{d}(t) = \vec{v}_\mathrm{d} \cdot t + \vec{d}_0 \tag{S24}$$

Next, we insert the linear approximation for the drift offset $\vec{d}$ (Equation (S24)) into the relation between the scanner and surface coordinates (Equation (S21)) to arrive at Equation (S25). In Equation (S25), the only quantity that is unknown at first is the drift velocity $\vec{v}_\mathrm{d}$. The scanner position $\vec{r}$ and time t are defined by the measurement procedure, and the drift offset can be chosen arbitrarily for drift correction of the first image in a measurement. For instance, we can define that the scanner and surface coordinates coincide at the beginning of a measurement session, that is, the initial drift offset is zero. Then, the drift offset for all subsequent images is given by the previous drift velocities. Hence, we can apply Equation (S25) to drift-correct SPM data with a known drift velocity.

$$\vec{r}_\mathrm{surf}(t) = \vec{r}(t) - \vec{v}_\mathrm{d}(t = 0) \cdot t - \vec{d}_0 \tag{S25}$$

Equation (S25) is very suitable for the pixel-wise correction of SPM data. For full SPM images, however, it is more efficient to employ a drift correction based on an affine transformation of pixel positions [15,18,29,32]. Hence, we will rewrite Equation (S25) to derive the affine transformation behind linear drift. Equation (S25) contains the scanner position $\vec{r}$ and time t as two separate parameters, but in reality these two quantities are linked by the measurement procedure. At any given time, the scanner is at exactly one position and, more importantly, for most raster scanning motions, the scanner visits each position exactly once per image [12,15,32]. Hence, we can choose whether we want to write the scanner position as a function of time or the time as a function of the scanner position [32]. The latter possibility enables us to drift-correct SPM images based on positions solely, which is needed for the derivation of an affine transformation [12,15].

For a linear scanning motion in x and y directions, the measurement time t is a linear function of the scanner location $\vec{r}$ given by Equation (S26) [12,15]. Here, τ_s is an auxiliary vector we refer to as time mapping vector, and $t_{s,0}$ is an offset time. The index s denotes that the time mapping is depending on the scan direction, which governs the order of measurement. We describe the derivation of the time mapping for all four standard scan directions with and without over-scanning in section 2.2.

$$t(\vec{r}) = \vec{\tau}_s * \vec{r} + t_{s,0} \tag{S26}$$

The offset time $t_{s,0}$ is defined by the measurement time and position of the first pixel. The first pixel is measured at $t = 0$ at the offset position $\vec{r}_s(0)$, which can be inserted into Equation (S26) and rearranged to yield Equation (S27) for the offset time. Note that the drift offset $\vec{r}_s(0)$, again, depends on the scan direction.

$$t_{s,0} = -\vec{\tau}_s * \vec{r}_s(0) \tag{S27}$$

Finally, we insert Equations (S26) and (S27) into Equation (S25) and rearrange the result to arrive at

Equations (S28) to (S30) for the inverse drift transformation $\hat{D}_s^{-1}$. The inverse drift transformation $\hat{D}_s^{-1}$ inverts the effect of drift on positions measured by SPM by transforming from the scanner coordinate system into surface coordinates. Here, $\hat{D}_s^{-1}$ is an affine transformation comprising the transformation matrix $\boldsymbol{D}_s^{-1}$ given by Equation (S29) and the offset vector $\vec{d}_s^{-1}$ given by Equation (S30) [15]. Both components of the inverse drift transformation only depend on the drift velocity $\vec{v}_\mathrm{d}$ and quantities governed by the scan direction denoted with an index s. Hence, we only need to measure the drift velocity to be able to drift-correct SPM data with Equation (S28). We discuss strategies to determine the drift velocity based on two consecutive SPM images in section 2.3.

$$\vec{r}_\mathrm{surf}(t) = \hat{D}_s^{-1}\vec{r} = \boldsymbol{D}_s^{-1}\vec{r} + \vec{d}_s^{-1} \tag{S28}$$

$$\boldsymbol{D}_s^{-1} = \mathbf{1} - \vec{v}_\mathrm{d}\vec{\tau}_s^\intercal \tag{S29}$$

$$\vec{d}_s^{-1} = \vec{v}_\mathrm{d}\vec{\tau}_s^\intercal \vec{r}_s(0) - \vec{d}_0 \tag{S30}$$

2.2 Time Mapping

The time mapping vector $\vec{\tau}_s$ is an essential part of the drift transformation, as it encodes the measurement times and, thus, the measurement order of pixels as governed by the scan direction s. Here, we derive the time mapping vectors and corresponding drift origins for two types of scanning procedures commonly applied in scanning probe microscopy, namely regular scanning and over-scanning procedures (see Figure S3). To this end, we show the derivation of $\vec{\tau}_s$ for one example scan direction; then, we give the results for all other scan directions.

For both scanning procedures presented here, the derivation of the time mapping vector is much easier

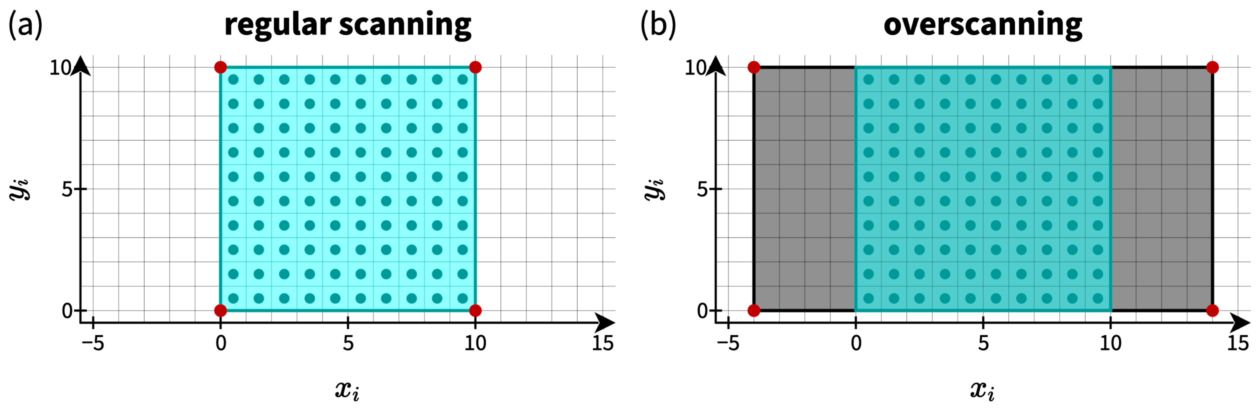

Figure S3: Schematic representation of the scan area in case of regular scanning (a) and over-scanning (b). In both cases, the scan area is shown as a cyan square, and the positions of the raster points are displayed as cyan dots. The over-scanning area, where no data is recorded, is shaded in gray. Corner points of the area of movement of the piezo scanner are shown in red.

in image coordinates than in scanner coordinates because of the alignment between basis vectors and scan directions in image coordinates. Hence, we will derive the time mapping vector and drift origin in image coordinates first and transform into the more complex scanner coordinates afterwards. To this end, we now discuss the transformation of the time mapping from image coordinates into scanner coordinates. Analogous to the description in scanner coordinates, the time mapping in image coordinates is given by Equation (S31). There, $\vec{r}_\mathrm{i}$ is the position vector in image coordinates, $\vec{\tau}_{\mathrm{i},s}$ is the time mapping vector in image coordinates, and $\vec{r}_{\mathrm{i},s}(0)$ is the corresponding drift origin.

$$t(\vec{r}_\mathrm{i}) = \vec{\tau}_{\mathrm{i},s} * \vec{r}_\mathrm{i} - \vec{\tau}_{\mathrm{i},s} * \vec{r}_{\mathrm{i},s}(0) \tag{S31}$$

To transform the image time mapping into scanner coordinates, we insert the transformation from scanner coordinates into image coordinates (Equation (S32)) into Equation (S31) and rearrange the result to obtain Equation (S33).

$$\vec{r}_\mathrm{i} = \boldsymbol{K}^{-1}\vec{r} - \boldsymbol{K}^{-1}\vec{r}_\mathrm{off} \tag{S32}$$

$$t(\vec{r}) = \vec{\tau}_{\mathrm{i},s}^{\mathsf{T}}\boldsymbol{K}^{-1}\vec{r} - \vec{\tau}_{\mathrm{i},s}^{\mathsf{T}}\boldsymbol{K}^{-1}\vec{r}_s(0) \tag{S33}$$

Comparison between Equation (S33) and Equation (S26) reveals that the time mapping vector $\vec{\tau}_s$ and drift origin $\vec{r}_s(0)$ in scanner coordinates are connected to their counterparts in image coordinates by Equations (S34) and (S35), respectively.

$$\vec{\tau}_s = (\boldsymbol{K}^{-1})^{\mathsf{T}}\vec{\tau}_{\mathrm{i},s} \tag{S34}$$

$$\vec{r}_s(0) = \boldsymbol{K}\vec{r}_{\mathrm{i},s}(0) + \vec{r}_\mathrm{off} \tag{S35}$$

2.2.1 Regular Scanning

Next, we derive the time mapping in image coordinates for the regular scanning shown in Figure S3a on the example of a trace-up image. In image coordinates, the scan directions are (pseudo) orthogonal and align with the basis vectors. Thus, we can discuss the time mapping in fast and slow scan directions separately and sum up the results. In fast scan direction, the scanner needs a time of $T/(2M)$ to travel from the first to the last pixel of each line, where T is the total measurement time per image and M the number of lines. Here, the factor of two encodes that the scanner moves along each line twice, once in trace and once in retrace direction. Moreover, the scanner travels the width of the image W once per line, so the x component of $\vec{\tau}_{\mathrm{i},\rightarrow\uparrow}$ is given by $T/(2MW)$. In slow scan direction, the scanner

needs the total measurement time T to travel the full image height H, so the y component of $\vec{\tau}_{\mathrm{i},\rightarrow\uparrow}$ is T/H. As the measurement in trace-up images is started in the origin of the image coordinate system, the drift origin coincides with the origin of the coordinate system. The results for the time mapping in image coordinates of all four possible scan directions are listed in Table S2. Note that the elements of the time mapping vector derived here are identical with the transformation derived by Woodward and Schwartz [15].

Table S2: Image-coordinate time mapping vectors $\vec{\tau}_{\mathrm{i},s}$ and drift origins $\vec{r}_{\mathrm{i},s}(0)$ for all four scan directions commonly applied during SPM measurements.

scan direction s	time mapping vector $\vec{\tau}_{\mathrm{i},s}$	drift origin $\vec{r}_{\mathrm{i},s}(0)$
trace up ($\rightarrow\uparrow$)	$\vec{\tau}_{\mathrm{i},\rightarrow\uparrow} = \begin{pmatrix} \frac{1}{2M}\frac{T}{W} \\ \frac{T}{H} \end{pmatrix}$	$\vec{r}_{\mathrm{i},\rightarrow\uparrow}(0) = \begin{pmatrix} 0 \\ 0 \end{pmatrix}$
retrace up ($\leftarrow\uparrow$)	$\vec{\tau}_{\mathrm{i},\leftarrow\uparrow} = \begin{pmatrix} -\frac{1}{2M}\frac{T}{W} \\ \frac{T}{H} \end{pmatrix}$	$\vec{r}_{\mathrm{i},\leftarrow\uparrow}(0) = \begin{pmatrix} W \\ 0 \end{pmatrix}$
trace down ($\rightarrow\downarrow$)	$\vec{\tau}_{\mathrm{i},\rightarrow\downarrow} = \begin{pmatrix} \frac{1}{2M}\frac{T}{W} \\ -\frac{T}{H} \end{pmatrix}$	$\vec{r}_{\mathrm{i},\rightarrow\downarrow}(0) = \begin{pmatrix} 0 \\ H \end{pmatrix}$
retrace down ($\leftarrow\downarrow$)	$\vec{\tau}_{\mathrm{i},\leftarrow\downarrow} = \begin{pmatrix} -\frac{1}{2M}\frac{T}{W} \\ -\frac{T}{H} \end{pmatrix}$	$\vec{r}_{\mathrm{i},\leftarrow\downarrow}(0) = \begin{pmatrix} W \\ H \end{pmatrix}$

2.2.2 Over-scanning

Some scanning probe microscopes use a different scanning procedure called over-scanning to minimize the effect of piezo hysteresis. In the case of over-scanning, the scanner not only passes the scan area where the SPM data is recorded as shown in Figure S3a but exceeds it in the x direction as shown in Figure S3b. This minimizes the distortion of the recorded SPM data, because it excludes the area near the turning points of the scanning motion, where the gradient of piezo hysteresis is bigger [7,9-11]. The time mapping is altered by overscanning due to the bigger area of scanner motion. This, however, can be included in the time mapping derived for regular scanning very easily. Compared to the regular scanning, the decisive width is no longer the image width W but the image width plus the width of over-scanning at the left and right edges of the image given by $W + 2O_x$. Moreover, the corner points of the scanning motion are now outside of the measurement area, so the

drift origin needs to be adapted as well. Considering both, we arrive at the time mapping vectors and drift offsets given in Table S3.

Table S3: Image-coordinate time mapping vectors $\vec{\tau}_{\mathrm{i},s}$ and drift origins $\vec{r}_{\mathrm{i},s}(0)$ for all four scan directions commonly applied during SPM measurements in case of over-scanning.

scan direction s	time mapping vector $\vec{\tau}_{\mathrm{i},s}$	drift origin $\vec{r}_{\mathrm{i},s}(0)$
trace up (→↑)	$\vec{\tau}_{\mathrm{i},\rightarrow\uparrow} = \begin{pmatrix} \frac{1}{2M}\frac{T}{W+2O_x} \\ (1-\frac{1}{M})\frac{T}{H} \end{pmatrix}$	$\vec{r}_{\mathrm{i},\rightarrow\uparrow}(0) = \begin{pmatrix} -O_x \\ 0 \end{pmatrix}$
retrace up (←↑)	$\vec{\tau}_{\mathrm{i},\leftarrow\uparrow} = \begin{pmatrix} -\frac{1}{2M}\frac{T}{W+2O_x} \\ (1-\frac{1}{M})\frac{T}{H} \end{pmatrix}$	$\vec{r}_{\mathrm{i},\leftarrow\uparrow}(0) = \begin{pmatrix} W+O_x \\ 0 \end{pmatrix}$
trace down (→↓)	$\vec{\tau}_{\mathrm{i},\rightarrow\downarrow} = \begin{pmatrix} \frac{1}{2M}\frac{T}{W+2O_x} \\ -(1-\frac{1}{M})\frac{T}{H} \end{pmatrix}$	$\vec{r}_{\mathrm{i},\rightarrow\downarrow}(0) = \begin{pmatrix} -O_x \\ H \end{pmatrix}$
retrace down (←↓)	$\vec{\tau}_{\mathrm{i},\leftarrow\downarrow} = \begin{pmatrix} -\frac{1}{2M}\frac{T}{W+2O_x} \\ -(1-\frac{1}{M})\frac{T}{H} \end{pmatrix}$	$\vec{r}_{\mathrm{i},\leftarrow\downarrow}(0) = \begin{pmatrix} W+O_x \\ H \end{pmatrix}$

2.3 Determination of the Drift Velocity

In the previous section, we show that the effect of a constant drift velocity can be easily corrected if the drift velocity $\vec{v}_{\mathrm{d}}$ is known. Next, we discuss how the drift velocity can be determined based on (1) the different distortion of periodic structures depending on the imaging direction and (2) the apparent movement of single stationary features or structures. All presented methods rely on the evaluation of two consecutive SPM images and only require information contained within the measured SPM data. They do not require any additional information on the surface structure such as the lattice parameters or surface reconstruction.

2.3.1 Distortion of the Surface Periodicity

For images exhibiting periodic structures, the drift velocity can be evaluated based on the distortion of these structures [12,15,29]. According to the drift transformations derived in section 2.1, a two-dimensional periodic structure as shown in Figure S4a will be distorted differently depending on the scan direction [15,29]. In our schematic example in Figure S4, we observe that the periodic structure is sheared in positive x direction and stretched along the y axis in the simulated up image

(Figure S4b). The periodic structure in the down image (Figure S4c), in contrast, is distorted in the opposite direction, that is, it is sheared in negative x direction and compressed in y direction. We can analyze this difference in the distortion depending on the scan direction to extract the drift velocity causing the image distortion.

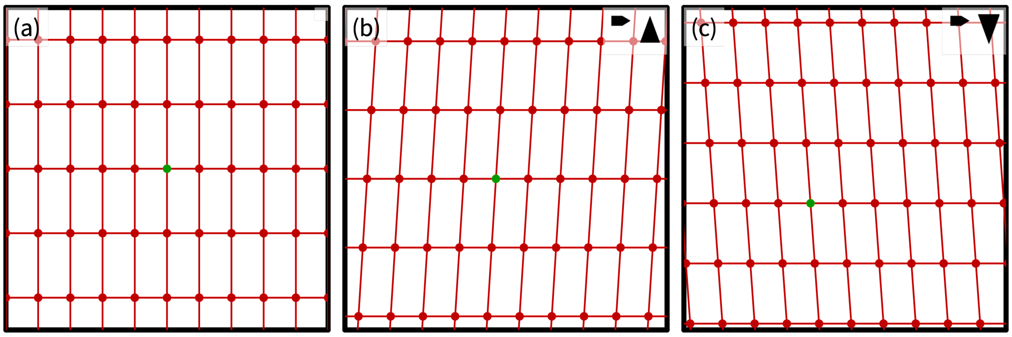

Figure S4: Schematic representation of the effect of drift on periodic structures. (a) shows a not distorted periodic structure, and (b) and (c) show the different distortions of these structures in up and down images. One lattice position is marked in all three images to show that it is not easily possible to decide which lattice positions correspond to each other in the images.

As the real surface periodicity is independent of the scan direction, the not distorted lattice vectors calculated from the apparent lattice vectors observed in two images need to be identical. This is shown in Equation (S36) for the two lattice vectors $\vec{a}_{\uparrow}$ and $\vec{a}_{\downarrow}$ extracted from one up and one down image. Here, it is important to note that $\vec{a}_{\uparrow}$ and $\vec{a}_{\downarrow}$ are directional vectors; thus, they are only affected by the linear part of the inverse drift transformation and not by the constant offset [12,15].

$$\vec{a} = \boldsymbol{D}_{\uparrow}^{-1}\vec{a}_{\uparrow} = \boldsymbol{D}_{\downarrow}^{-1}\vec{a}_{\downarrow} \tag{S36}$$

Next, we insert the functional form of the inverse drift matrix $\boldsymbol{D}_i^{-1}$ (Equation (S29)) into Equation (S36) and solve it for the drift velocity $\vec{v}_{\mathrm{d}}$ to arrive at Equation (S37). For the second lattice vector $\vec{b}$, we can derive the corresponding expression for the drift velocity given by Equation (S38).

$$\vec{v}_{\mathrm{d}} = \frac{\vec{a}_{\uparrow} - \vec{a}_{\downarrow}}{\vec{\tau}_{\uparrow}\vec{a}_{\uparrow} - \vec{\tau}_{\downarrow}\vec{a}_{\downarrow}} \tag{S37}$$

$$\vec{v}_{\mathrm{d}} = \frac{\vec{b}_{\uparrow} - \vec{b}_{\downarrow}}{\vec{\tau}_{\uparrow}\vec{b}_{\uparrow} - \vec{\tau}_{\downarrow}\vec{b}_{\downarrow}} \tag{S38}$$

Based on Equations (S37) and (S38), we can calculate the drift velocity from the apparent lattice vectors extracted from two SPM images with different scan direction. This method does not work for images measured in the same scan direction, because in that case we would observe the same apparent lattice vectors in both images. Moreover, it is crucial for this method to use apparent vectors that originate from distortion of the same vector in surface coordinates. Especially for high drift velocities, this selection of corresponding vectors is not always straightforward, which is why we present an algorithm to solve this problem in the main text.

In principle, this method is not limited to the lattice vectors of a two-dimensional periodic structure but can be applied to any vector that can be identified in two images measured with different scan directions. However, it can be challenging to find matching pairs of vectors as described above.

2.3.2 Stationary Features and Structures

Next, we discuss the evaluation of drift velocities based on the apparent movement of stationary features or structures [14,16,18-20]. As their name suggests, stationary features and structures are objects on the sample surface that do not change their position or shape on the timescale of the experiment. Typical examples for stationary features observed in SPM images are defects, step edges, or islands that do not change their shape during the measurement time. As stationary features stay fixed on the surface, they should not move in SPM images. This, however, is no longer the case if the measurement is affected by drift. The drift moves the scanner relative to the surface; thus, stationary features seem to move in the SPM images [14,16,18-20]. We show the principle of this

effect in Figure S2b and Figure S5. Figure S2b shows the scan areas of three consecutive images in surface coordinates and the positions of four stationary features in different colors. It is evident that the relative positions in the scan areas are different for all three images despite the features staying fixed on the surface. This apparent movement becomes even clearer, when we look at the corresponding schematic SPM images shown in Figure S5. Here, it is especially important to note that the apparent shift of features between two images also depends on the scan directions of these images [18]. Figure S5a and Figure S5b are simulated with opposing slow scan directions, and the apparent shift is different for each feature depending on its position in the image. For example, the position of the yellow feature stays nearly the same, while the red feature shifted significantly. When we compare Figure S5a and Figure S5c, this is different. All features are shifted by the same distance, regardless of their position in the image. This is a general effect caused by the different distortion of SPM images by drift depending on the scan direction as shown in section 2.1. Figure S5a and Figure S5c are both trace-up images and, thus, distorted in the same way. Hence, all features are shifted by the same vector. Figure S5b, in contrast, is a trace-down image, which is why it is distorted differently and the apparent shift of features is different depending on their position (see Figure S2b).

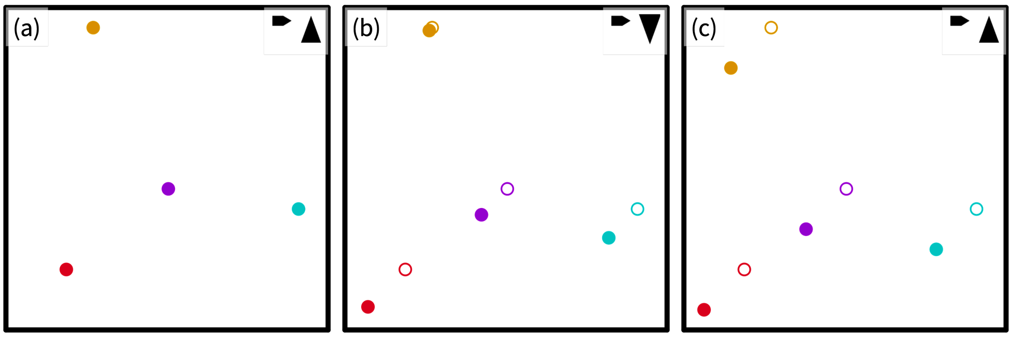

Figure S5: Schematic representation of the apparent shift of stationary features in SPM images affected by drift. Shown are three consecutive images with alternating scan directions. (a) and (c) are simulated with trace-up direction and (b) with trace-down direction. The positions of four exemplary stationary features are shown in all three images as differently colored filled circles. To show the effect of drift, the positions of the features in (a) are shown in (b) and (c) as open circles in the same colors. The scan areas in surface coordinates corresponding to images (a), (b), and (c) and the surface positions of the shown stationary features are shown in Figure S2.

To evaluate the drift velocity based on this apparent shift of stationary features, we now turn to the

mathematical description. As stationary features do not change their position on the surface, the surface positions of one feature derived from two different images need to be the same [18,20]. This consideration leads to Equation (S39), where $\vec{r}_{\mathrm{surf},1}$ and $\vec{r}_{\mathrm{surf},2}$ are the positions of the stationary feature derived from image one and two, respectively.

$$\vec{r}_{\mathrm{surf},1} = \vec{r}_{\mathrm{surf},2} \tag{S39}$$

In Equation (S39), we can replace the surface positions $\vec{r}_{\mathrm{surf},i}$ by the position in scanner coordinates and the components of the inverse drift transformation according to Equation (S28) to achieve Equation (S40).

$$\vec{r}_2 - \vec{r}_1 = \vec{v}_\mathrm{d}\,[\vec{\tau}_2(\vec{r}_2 - \vec{r}_2(0)) - \vec{\tau}_1(\vec{r}_1 - \vec{r}_1(0))] + \vec{d}_{0,2} - \vec{d}_{0,1} \tag{S40}$$

Next, we evaluate the difference between the drift offsets $\vec{d}_{0,2} - \vec{d}_{0,1}$. These offsets describe the accumulated shift between the surface and the scanner coordinate systems at starting times of images one and two, respectively. Given that the drift velocity is constant, which is one of our basis assumptions, this difference in drift offset can be expressed as the time Δt that passed between the starts of images one and two multiplied by the drift velocity $\vec{v}_\mathrm{d}$. We insert this expression in Equation (S40) and solve for the drift velocity to arrive at Equation (S41).

$$\vec{v}_\mathrm{d} = \frac{\vec{r}_2 - \vec{r}_1}{\vec{\tau}_2(\vec{r}_2 - \vec{r}_2(0)) - \vec{\tau}_1(\vec{r}_1 - \vec{r}_1(0)) + \Delta t} \tag{S41}$$

For two images measured in the same scan direction, the τ_i and $\vec{r}_i(0)$ components of the drift transformation are identical (see Table S2). Hence, Equation (S41) can be simplified to Equation (S42) [20]. This equation does not depend on the positions of the feature in both images anymore, but only

on the apparent position shift between both images $\Delta\vec{r} = \vec{r}_2 - \vec{r}_1$.

$$\vec{v}_{\mathrm{d}} = \frac{\vec{r}_2 - \vec{r}_1}{\vec{\tau}(\vec{r}_2 - \vec{r}_1) + \Delta t} = \frac{\Delta\vec{r}}{\vec{\tau}\Delta\vec{r} + \Delta t} \tag{S42}$$

Next, we want to discuss the equations derived for the calculation of the drift velocity from the movement of stationary features. With Equation (S41), we can calculate the drift velocity from the positions observed for one stationary feature in two consecutive SPM images regardless of the scan direction. Equation (S42), in contrast, only applies to two images recorded in the same scan direction. In this sense, it seems that Equation (S41) is superior to Equation (S42). However, Equation (S42) opens an additional possibility to evaluate the drift velocity, as it only depends on the shift of features $\Delta\vec{r}$ and not on their positions. As the shift of stationary features between two images measured with the same scan direction is the same for all features, this shift can be extracted very easily by analyzing the cross-correlation image as described in [14,16,36]. We also discuss this method in the main text of our publication. In this regard, it is important to keep in mind that this cross-correlation method only applies to images recorded in the same scan direction.

3 Experimental Methods

3.1 AFM in Ultrahigh Vacuum

Dynamic atomic force microscopy (dAFM) measurements were carried out in an ultrahigh vacuum (UHV) chamber with an VT STM/AFM device (Scienta Omicron, Taunusstein, Germany) operated by a MATRIX controller. For the measurements, N-doped silicon cantilevers with a nominal force constant of $40\,\mathrm{N\,m^{-1}}$ and an eigenfrequency of 300 kHz were glued onto a cantilever holder, brought into vacuum and cleaned via Ar-ion bombardment. dAFM experiments were performed in the frequency modulation mode with constant amplitudes between 5 nm and 20 nm (zero-peak). During the measurements, a bias voltage between -10 V and 10 V was applied to compensate for the contact voltage of the sample. Low-temperature experiments were performed under liquid nitrogen cooling with a flow cryostat. The base pressure during the AFM measurements was below 3×10^{-11} mbar.

Oriented calcium fluoride single crystals were degassed in UHV and cleaved in situ parallel to the (111) crystal direction. After cleavage, the sample crystals were annealed in a manipulator with a PBN heater at 2 h for 415 K. Oriented calcite single crystals (Korth Kristalle GmbH, Germany) were degassed, cleaved parallel to the (10.4) crystal plane and annealed for 4 h. The preparation was performed in a part of the UHV chamber that can be separated from the AFM chamber, and the pressure during sample preparation did not exceed 2×10^{-9} mbar.

3.2 AFM at the Solid–Liquid Interface

AFM measurements with atomic resolution of the calcite(10.4)–water interface were performed over a measurement time of up to 7 h without changing the scan parameters. Two different AFMs were used for these measurements: a Cypher ES AFM (Asylum Research, an Oxford Instrument Company, USA) and a modified [41,42] Bruker Multimode with a Nanoscope V Controller (Bruker Nano Surface Division, USA). Both AFMs operated in the frequency modulation mode and are equipped with a laser for photothermal excitation. For both AFMs, back side gold-coated silicon cantilevers (TAP300GD-G, Budget Sensors, Bulgaria) with a nominal force constant of $40\,\mathrm{N\,m^{-1}}$ were used. In water, the eigenfrequency of these cantilevers ranges from 108 kHz to 138 kHz, and the quality factor is between 6.5 and 8.5. The surface crystal orientation of the calcite samples (Korth Kristalle GmbH, Germany) was determined using the birefringence of the used calcite crystals. The calcite samples were oriented along the $[42\bar{1}]$ direction and clamped into a sample holder. Subsequently, the samples were cleaved along the (10.4) plane with a razor blade. A drop of ultra pure water (Stakpure GmbH, Germany, 18.3 MΩ cm) was placed on the freshly cleaved calcite sample.

3.3 Legend in AFM Images

In all AFM images, the fast and slow scan directions are marked with an arrow and a triangle in the top right corner, respectively. The channel displayed in the images is indicated with z_p for the z piezo displacement and $\Delta\nu_\mathrm{exc}$ for the excitation frequency shift.

4 Additional Experimental Data

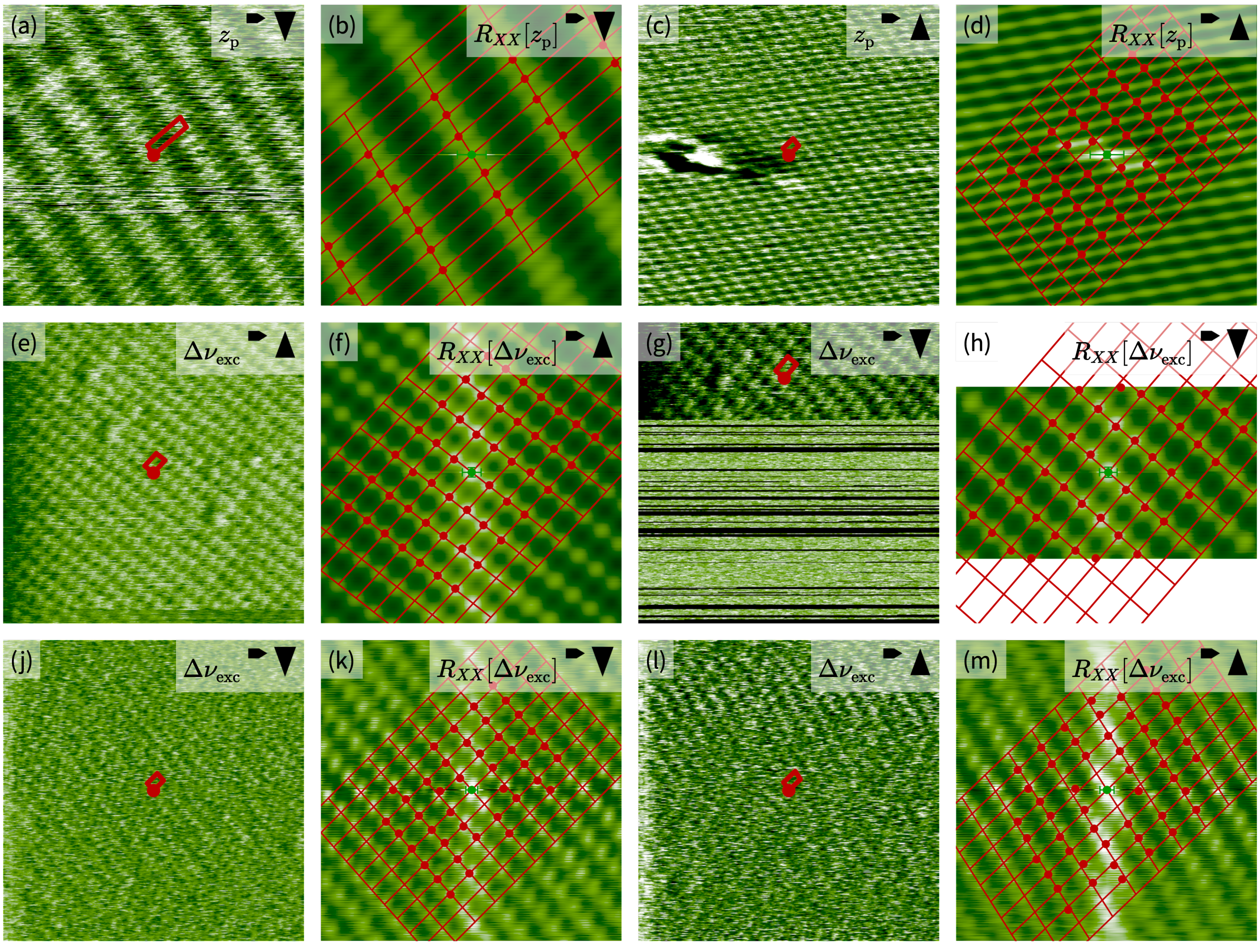

Figure S6: Raw-data images (first and third column) and corresponding autocorrelation images (second and fourth column) for the examples discussed in Figure 4 of the main text. The example images show SPM experiments with high drift velocities (a–d), only partly usable images (e–h), and a bad image quality (j–m). In all real-space images, the unit cell used for drift correction is shown as a red quadrangle. Images (a–d) show the atomic structure of calcium fluoride (111) recorded in UHV, and (e–h) and (j–m) were recorded at the calcite(10.4)–water interface. The maxima in the autocorrelation images are marked by red circles, and the optimized lattices as found by *un*Drift are shown as red lines. Only the centers of the autocorrelations are shown.

5 Author Contributions

TD developed and tested the *un*Drift software and underlying mathematical models. FS performed the AFM experiments at the solid–liquid interface, and TD and LH carried out AFM experiments

in ultrahigh vacuum. FS and TD analyzed the experimental data. TD wrote the first versions of manuscript and Supporting Information with contributions from AK, FS, and RB. All authors commented the manuscript and contributed to the final version of the manuscript. All authors agreed upon the final version of the manuscript.

References

1. Adler, J. G.; Chen, T. T.; Gallagher, M. C.; Konkin, M. K.; Mullin, D. P. *J. Vac. Sci. Technol., B: Microelectron. Nanometer Struct.–Process., Meas., Phenom.* **1991**, *9*, 992. doi:10.1116/1.585443.

2. Cai, C. Z.; Chen, X. Y.; Shu, Q. Q.; Zheng, X. L. *Rev. Sci. Instrum.* **1992**, *63*, 5649–5652. doi:10.1063/1.1143395.

3. Carrara, S.; Facci, P.; Nicolini, C. *Rev. Sci. Instrum.* **1994**, *65*, 2860–2863. doi:10.1063/1.1145207.

4. Staub, R.; Alliata, D.; Nicolini, C. *Rev. Sci. Instrum.* **1995**, *66*, 2513–2516. doi:10.1063/1.1145650.

5. Korpelainen, V.; Lassila, A. *Meas. Sci. Technol.* **2007**, *18*, 395–403. doi:10.1088/0957-0233/18/2/s11.

6. Marinello, F.; Savio, E. *Meas. Sci. Technol.* **2007**, *18*, 462–468. doi:10.1088/0957-0233/18/2/s19.

7. Jorgensen, J. F.; Carneiro, K.; Madsen, L. L.; Conradsen, K. *J. Vac. Sci. Technol., B: Microelectron. Nanometer Struct.–Process., Meas., Phenom.* **1994**, *12*, 1702. doi:10.1116/1.587267.

8. Trawick, M. L.; Megens, M.; Angelescu, D. E.; Harrison, C.; Vega, D. A.; Chaikin, P. M.; Register, R. A.; Adamson, D. H. *Scanning* **2003**, *25*, 25–33. doi:10.1002/sca.4950250106.

9. Stoll, E. P. *Rev. Sci. Instrum.* **1994**, *65*, 2864–2869. doi:10.1063/1.1144629.

10. Libioulle, L.; Ronda, A.; Taborelli, M.; Gilles, J. M. *J. Vac. Sci. Technol., B: Microelectron. Nanometer Struct.–Process., Meas., Phenom.* **1991**, *9*, 655. doi:10.1116/1.585480.

11. van de Leemput, L. E. C.; Rongen, P. H. H.; Timmerman, B. H.; van Kempen, H. *Rev. Sci. Instrum.* **1991**, *62*, 989–992. doi:10.1063/1.1141989.

12. Jorgensen, J. F.; Madsen, L. L.; Garnaes, J.; Carneiro, K.; Schaumburg, K. *J. Vac. Sci. Technol., B: Microelectron. Nanometer Struct.–Process., Meas., Phenom.* **1994**, *12*, 1698. doi:10.1116/1.587266.

13. Yurov, V. Y.; Klimov, A. N. *Rev. Sci. Instrum.* **1994**, *65*, 1551–1557. doi:10.1063/1.1144890.

14. Sollböhmer, O.; May, K.-P.; Anders, M. *Thin Solid Films* **1995**, *264*, 176–183. doi:10.1016/0040-6090(95)05847-8.

15. Woodward, J. T.; Schwartz, D. K. *J. Vac. Sci. Technol., B: Microelectron. Nanometer Struct.–Process., Meas., Phenom.* **1998**, *16*, 51. doi:10.1116/1.589834.

16. Mantooth, B. A.; Donhauser, Z. J.; Kelly, K. F.; Weiss, P. S. *Rev. Sci. Instrum.* **2002**, *73*, 313–317. doi:10.1063/1.1427417.

17. Sun, Y.; Pang, J. H. L. *Nanotechnology* **2006**, *17*, 933–939. doi:10.1088/0957-4484/17/4/016.

18. Lapshin, R. V. *Meas. Sci. Technol.* **2007**, *18*, 907–927. doi:10.1088/0957-0233/18/3/046.

19. Clifford, C. A.; Seah, M. P. *Meas. Sci. Technol.* **2009**, *20*, 095103. doi:10.1088/0957-0233/20/9/095103.

20. Rahe, P.; Bechstein, R.; Kühnle, A. *J. Vac. Sci. Technol., B: Microelectron. Nanometer Struct.–Process., Meas., Phenom.* **2010**, *28*, C4E31–C4E38. doi:10.1116/1.3360909.

21. Salmons, B. S.; Katz, D. R.; Trawick, M. L. *Ultramicroscopy* **2010**, *110*, 339–349. doi:10.1016/j.ultramic.2010.01.006.

22. Marinello, F.; Balcon, M.; Schiavuta, P.; Carmignato, S.; Savio, E. *Meas. Sci. Technol.* **2011**, *22*, 094016. doi:10.1088/0957-0233/22/9/094016.

23. Niu, D.; Chen, Y.; Huang, W. *Anal. Sci.* **2011**, *27*, 149–152. doi:10.2116/analsci.27.149.

24. Follin, N. D.; Taylor, K. D.; Musalo, C. J.; Trawick, M. L. *Rev. Sci. Instrum.* **2012**, *83*, 083711. doi:10.1063/1.4738646.

25. Degenhardt, J.; Tutsch, R.; Dai, G. *Meas. Sci. Technol.* **2021**, *32*, 035005. doi:10.1088/1361-6501/abc9f8.

26. Kizu, R.; Misumi, I.; Hirai, A.; Gonda, S. *Meas. Sci. Technol.* **2020**, *31*, 054009. doi:10.1088/1361-6501/ab6b50.

27. Sun, X.; Heaps, E.; Yacoot, A.; Yang, Q.; Grolich, P.; Klapetek, P. *Meas. Sci. Technol.* **2021**, *32*, 115010. doi:10.1088/1361-6501/ac100f.

28. Diao, Z.; Ueda, K.; Hou, L.; Yamashita, H.; Custance, O.; Abe, M. *Appl. Phys. Lett.* **2023**, *122*, 121601. doi:10.1063/5.0139330.

29. Henriksen, K.; Stipp, S. L. S. *Am. Mineral.* **2002**, *87*, 5–16. doi:10.2138/am-2002-0102.

30. Zhang, L.; Long, Q.; Liu, Y.; Zhang, J.; Feng, Z. *Ultramicroscopy* **2016**, *166*, 16–26. doi:10.1016/j.ultramic.2016.03.013.

31. Poirier, G. E.; White, J. M. *Rev. Sci. Instrum.* **1990**, *61*, 3917–3918. doi:10.1063/1.1141526.

32. Yothers, M. P.; Browder, A. E.; Bumm, L. A. *Rev. Sci. Instrum.* **2017**, *88*, 013708. doi:10.1063/1.4974271.

33. Fu, J.; Chu, W.; Dixson, R.; Orji, G.; Vorburger, T.; Secula, E. M.; Seiler, D. G.; Khosla, R. P.; Herr, D.; Garner, C. M.; McDonald, R.; Diebold, A. C. *AIP Conf. Proc.* **2009**, *1173*, 280–284. doi:10.1063/1.3251234.

34. Zhang, L.; Chen, X.; Huang, J.; Li, H.; Chen, L.; Huang, Q. *Rev. Sci. Instrum.* **2019**, *90*, 023704. doi:10.1063/1.5052281.

35. Gómez-Rodríguez, J. M.; Sáenz, J. J.; Baró, A. M.; Veuillen, J.-Y.; Cinti, R. C. *Phys. Rev. Lett.* **1996**, *76*, 799–802. doi:10.1103/physrevlett.76.799.

36. Huang, W.; Wang, W.; Xia, A.; Jin, N.; Hu, Z. *J. Vac. Sci. Technol., B: Microelectron. Nanometer Struct.–Process., Meas., Phenom.* **2000**, *18*, 2027. doi:10.1116/1.1306302.

37. Rahe, P.; Schütte, J.; Schniederberend, W.; Reichling, M.; Abe, M.; Sugimoto, Y.; Kühnle, A. *Rev. Sci. Instrum.* **2011**, *82*, 063704. doi:10.1063/1.3600453.

38. Niu, D.; Li, J.; Chen, Y.; Huang, W. *J. Vac. Sci. Technol., B: Microelectron. Nanometer Struct.–Process., Meas., Phenom.* **2010**, *28*, 1070–1072. doi:10.1116/1.3478305.

39. Gaponenko, I.; Tückmantel, P.; Ziegler, B.; Rapin, G.; Chhikara, M.; Paruch, P. *Sci. Rep.* **2017**, *7*, 669. doi:10.1038/s41598-017-00765-w.

40. Marinello, F.; Bariani, P.; Chiffre, L. D.; Savio, E. *Meas. Sci. Technol.* **2007**, *18*, 689–696. doi:10.1088/0957-0233/18/3/019.

41. Adam, H.; Rode, S.; Schreiber, M.; Kobayashi, K.; Yamada, H.; Kühnle, A. *Rev. Sci. Instrum.* **2014**, *85*, 023703. doi:10.1063/1.4864084.

42. Rode, S.; Stark, R.; Lübbe, J.; Tröger, L.; Schütte, J.; Umeda, K.; Kobayashi, K.; Yamada, H.; Kühnle, A. *Rev. Sci. Instrum.* **2011**, *82*, 073703. doi:10.1063/1.3606399.
